# Mechanical ventilation in patients with cardiogenic pulmonary edema: a sub-analysis of the LUNG SAFE study

**DOI:** 10.1186/s40560-022-00648-x

**Published:** 2022-12-25

**Authors:** Laura Amado-Rodríguez, Raquel Rodríguez-Garcia, Giacomo Bellani, Tài Pham, Eddy Fan, Fabiana Madotto, John G. Laffey, Guillermo M. Albaiceta, LUNG SAFE investigators, Antonio Pesenti, Laurent Brochard, Andres Esteban, Luciano Gattinoni, Frank van Haren, Anders Larsson, DanielF McAuley, Marco Ranieri, Gordon Rubenfeld, B. Taylor Thompson, Hermann Wrigge, Arthur S. Slutsky, Fernando Rios, Frank Van Haren, Thierry Sottiaux, Pieter Depuydt, Fredy S. Lora, Luciano Cesar Azevedo, Guillermo Bugedo, Haibo Qiu, Marcos Gonzalez, Juan Silesky, Vladimir Cerny, Jonas Nielsen, Manuel Jibaja, Hermann Wrigge, Dimitrios Matamis, Jorge Luis Ranero, Pravin Amin, S. M. Hashemian, Kevin Clarkson, Kiyoyasu Kurahashi, Asisclo Villagomez, Amine Ali Zeggwagh, Leo M. Heunks, Jon Henrik Laake, Jose Emmanuel Palo, Antero do Vale Fernandes, Dorel Sandesc, Yaasen Arabi, Vesna Bumbasierevic, Nicolas Nin, Jose A. Lorente, Anders Larsson, Lise Piquilloud, Fekri Abroug, DanielF McAuley, Lia McNamee, Javier Hurtado, Ed Bajwa, Gabriel Démpaire, Hektor Sula, Lordian Nunci, Alma Cani, Alan Zazu, Christian Dellera, Carolina S. Insaurralde, Sanatorio Las Lomas, San Isidro, Risso V. Alejandro, Julio Daldin, Mauricio Vinzio, Ruben O. Fernandez, Luis P. Cardonnet, Lisandro R. Bettini, Mariano Carboni Bisso, Emilio M. Osman, Mariano G. Setten, Pablo Lovazzano, Javier Alvarez, Veronica Villar, Norberto C. Pozo, Nicolas Grubissich, Gustavo A. Plotnikow, Daniela N. Vasquez, Santiago Ilutovich, Norberto Tiribelli, Ariel Chena, Carlos A. Pellegrini, María G. Saenz, Elisa Estenssoro, Matias Brizuela, Hernan Gianinetto, Pablo E. Gomez, Valeria I. Cerrato, Marco G. Bezzi, Silvina A. Borello, Flavia A. Loiacono, Adriana M. Fernandez, Serena Knowles, Claire Reynolds, Deborah M. Inskip, Jennene J. Miller, Jing Kong, Christina Whitehead, Shailesh Bihari, Aylin Seven, Amanda Krstevski, Helen J. Rodgers, Rebecca T. Millar, Toni E. McKenna, Irene M. Bailey, Gabrielle C. Hanlon, Anders Aneman, Joan M. Lynch, Raman Azad, John Neal, Paul W. Woods, Brigit L. Roberts, Mark R. Kol, Helen S. Wong, Katharina C. Riss, Thomas Staudinger, Xavier Wittebole, Caroline Berghe, Pierre A. Bulpa, Alain M. Dive, Rik Verstraete, Herve Lebbinck, Pieter Depuydt, Joris Vermassen, Philippe Meersseman, Helga Ceunen, JonasI Rosa, Daniel O. Beraldo, Claudio Piras, Adenilton M. Rampinelli, Antonio P. Nassar Jr, Sergio Mataloun, Marcelo Moock, MarlusM Thompson, Claudio H. Gonçalves, Ana Carolina P. Antônio, Aline Ascoli, Rodrigo S. Biondi, Danielle C. Fontenele, Danielle Nobrega, Vanessa M. Sales, Ahmad Yazid BinHJAbul Wahab, Maizatul Ismail, Suresh Shindhe, John Laffey, Francois Beloncle, Kyle G. Davies, Rob Cirone, Venika Manoharan, Mehvish Ismail, Ewan C. Goligher, Mandeep Jassal, Erin Nishikawa, Areej Javeed, Gerard Curley, Nuttapol Rittayamai, Matteo Parotto, Niall D. Ferguson, Sangeeta Mehta, Jenny Knoll, Antoine Pronovost, Sergio Canestrini, Alejandro R. Bruhn, Patricio H. Garcia, Felipe A. Aliaga, Pamela A. Farías, Jacob S. Yumha, Claudia A. Ortiz, Javier E. Salas, Alejandro A. Saez, Luis D. Vega, Eduardo F. Labarca, Felipe T. Martinez, Nicolás G. Carreño, Pilar Lora, Haitao Liu, Haibo Qiu, Ling Liu, Rui Tang, Xiaoming Luo, Youzhong An, Huiying Zhao, Yan Gao, Zhe Zhai, Zheng L. Ye, Wei Wang, Wenwen Li, Qingdong Li, Ruiqiang Zheng, Wenkui Yu, Juanhong Shen, Xinyu Li, Tao Yu, Weihua Lu, Ya Q. Wu, Xiao B. Huang, Zhenyang He, Yuanhua Lu, Hui Han, Fan Zhang, Renhua Sun, Hua X. Wang, Shu H. Qin, Bao H. Zhu, Jun Zhao, Jian Liu, Bin Li, Jing L. Liu, Fa C. Zhou, Qiong J. Li, Xing Y Zhang, Zhou Li-Xin, Qiang Xin-Hua, Liangyan Jiang, Yuan N. Gao, Xian Y. Zhao, Yuan Y. Li, Xiao L. Li, Chunting Wang, Qingchun Yao, Rongguo Yu, Kai Chen, Huanzhang Shao, Bingyu Qin, Qing Q. Huang, Wei H. Zhu, Ai Y. Hang, Ma X. Hua, Yimin Li, Yonghao Xu, Yu D. Di, Long L. Ling, Tie H. Qin, ShouH Wang, Junping Qin, Yi Han, Suming Zhou, Monica P. Vargas, Juan I. Silesky Jimenez, Manuel A. González Rojas, Jaime E. SolisQuesada, ChristianM Ramirez-Alfaro, Jan Máca, Peter Sklienka, Jakob Gjedsted, Aage Christiansen, Jonas Nielsen, BorisG Villamagua, Iguel Llano, Philippe Burtin, Gautier Buzancais, Pascal Beuret, Nicolas Pelletier, Satar Mortaza, Alain Mercat, Jonathan Chelly, Sébastien Jochmans, Nicolas Terzi, Cédric Daubin, Guillaume Carteaux, Nicolas de Prost, Jean-Daniel Chiche, Fabrice Daviaud, Muriel Fartoukh, Guillaume Barberet, Jerome Biehler, Jean Dellamonica, Denis Doyen, Jean-Michel Arnal, Anais Briquet, Sami Hraiech, Laurent Papazian, Arnaud Follin, Damien Roux, Jonathan Messika, Evangelos Kalaitzis, Laurence Dangers, Alain Combes, Gaetan Béduneau, Dorothée Carpentier, Elie H. Zogheib, Herve Dupont, Sylvie Ricome, FrancescoL Santoli, Sebastien L. Besset, Philippe Michel, Bruno Gelée, Pierre-Eric Danin, Bernard Goubaux, Philippe J. Crova, Nga T. Phan, Frantz Berkelmans, Julio C. Badie, Romain Tapponnier, Josette Gally, Samy Khebbeb, Jean-Etienne Herbrecht, Francis Schneider, PierreLouis M. Declercq, Jean-Philippe Rigaud, Jacques Duranteau, Anatole Harrois, Russell Chabanne, Julien Marin, Charlene Bigot, Sandrine Thibault, Mohammed Ghazi, Messabi Boukhazna, Salem Ould Zein, Jack R. Richecoeur, DanieleM Combaux, Fabien Grelon, Charlene Le Moal, EliseP Sauvadet, Adrien Robine, Virginie Lemiale, Danielle Reuter, Martin Dres, Alexandre Demoule, Dany Goldgran-Toledano, Loredana Baboi, Claude Guérin, Ralph Lohner, Jens Kraßler, Susanne Schäfer, Kai D. Zacharowski, Patrick Meybohm, Andreas W. Reske, Philipp Simon, HansBernd F. Hopf, Michael Schuetz, Thomas Baltus, Metaxia N. Papanikolaou, Theonymfi G. Papavasilopoulou, Giannis A. Zacharas, Vasilis Ourailogloy, Eleni K. Mouloudi, Eleni V. Massa, Eva O. Nagy, Electra E. Stamou, Ellada V. Kiourtzieva, Marina A. Oikonomou, Luis E. Avila, Cesar A. Cortez, Johanna E. Citalán, Sameer A. Jog, Safal D. Sable, Bhagyesh Shah, Mohan Gurjar, Arvind K. Baronia, Mohammedfaruk Memon, Radhakrishnan Muthuchellappan, Venkatapura J. Ramesh, Anitha Shenoy, Ramesh Unnikrishnan, Subhal B. Dixit, RachanaV Rhayakar, Nagarajan Ramakrishnan, VallishK Bhardwaj, HeeraL Mahto, Sudha V. Sagar, Vijayanand Palaniswamy, Deeban Ganesan, Seyed Mohammadreza Hashemian, Hamidreza Jamaati, Farshad Heidari, Edel A. Meaney, Alistair Nichol, Karl M. Knapman, Donall O’Croinin, EimhinS Dunne, Dorothy M. Breen, Kevin P. Clarkson, Rola F. Jaafar, Rory Dwyer, Fahd Amir, Olaitan O. Ajetunmobi, Aogan C. O’Muircheartaigh, ColinS Black, Nuala Treanor, Daniel V. Collins, Wahid Altaf, Gianluca Zani, Maurizio Fusari, Savino Spadaro, Carlo A. Volta, Romano Graziani, Barbara Brunettini, Salvatore Palmese, Paolo Formenti, Michele Umbrello, Andrea Lombardo, Elisabetta Pecci, Marco Botteri, Monica Savioli, Alessandro Protti, Alessia Mattei, Lorenzo Schiavoni, Andrea Tinnirello, Manuel Todeschini, Antonino Giarratano, Andrea Cortegiani, Sara Sher, Anna Rossi, Massimo M. Antonelli, Luca M. Montini, Paolo Casalena, Sergio Scafetti, Giovanna Panarello, Giovanna Occhipinti, Nicolò Patroniti, Matteo Pozzi, RobertoR Biscione, Michela M. Poli, Ferdinando Raimondi, Daniela Albiero, Giulia Crapelli, Eduardo Beck, Vincenzo Pota, Vincenzo Schiavone, Alexandre Molin, Fabio Tarantino, Giacomo Monti, Elena Frati, Lucia Mirabella, Gilda Cinnella, Tommaso Fossali, Riccardo Colombo, Pierpaolo Terragni, Ilaria Pattarino, Francesco Mojoli, Antonio Braschi, Erika E. Borotto, Andrea N. Cracchiolo, Daniela M. Palma, Francesco Raponi, Giuseppe Foti, EttoreR Vascotto, Andrea Coppadoro, Luca Brazzi, Leda Floris, Giorgio A. Iotti, Aaron Venti, Osamu Yamaguchi, Shunsuke Takagi, Hiroki N. Maeyama, Eizo Watanabe, Yoshihiro Yamaji, Kazuyoshi Shimizu, Kyoko Shiozaki, Satoru Futami, Sekine Ryosuke, Koji Saito, Yoshinobu Kameyama, Keiko Ueno, Masayo Izawa, Nao Okuda, Hiroyuki Suzuki, Tomofumi Harasawa, Michitaka Nasu, Tadaaki Takada, Fumihito Ito, Shin Nunomiya, Kansuke Koyama, Toshikazu Abe, Kohkichi Andoh, Kohei Kusumoto, Akira Hirata, Akihiro Takaba, Hiroyasu Kimura, Shuhei Matsumoto, Ushio Higashijima, Hiroyuki Honda, Nobumasa Aoki, Hiroshi Imai, Yasuaki Ogino, Ichiko Mizuguchi, Kazuya Ichikado, Kenichi Nitta, Katsunori Mochizuki, Tomoaki Hashida, Hiroyuki Tanaka, Tomoyuki Nakamura, Daisuke Niimi, Takeshi Ueda, Yozo Kashiwa, Akinori Uchiyama, Olegs Sabelnikovs, Peteris Oss, Youssef Haddad, Kong Y. Liew, Silvio A. Ñamendys-Silva, YvesD Jarquin-Badiola, Luis A. Sanchez-Hurtado, Saira S. Gomez-Flores, Maria C. Marin, AsiscloJ Villagomez, Jordana S. Lemus, Jonathan M. Fierro, Mavy Ramirez Cervantes, Francisco Javier Flores Mejia, Dulce Dector, Alejandro Rojas, Daniel R. Gonzalez, Claudia R. Estrella, Jorge R. Sanchez-Medina, Alvaro Ramirez-Gutierrez, Fernando G. George, Janet S. Aguirre, Juan A. Buensuseso, Manuel Poblano, Tarek Dendane, Amine Ali Zeggwagh, Hicham Balkhi, Mina Elkhayari, Nacer Samkaoui, Hanane Ezzouine, Abdellatif Benslama, Mourad Amor, Wajdi Maazouzi, Nedim Cimic, Oliver Beck, Monique M. Bruns, Jeroen A. Schouten, Myra Rinia, Monique Raaijmakers, Leo M. Heunks, Hellen M. Van Wezel, SergeJ Heines, Ulrich Strauch, Marc P. Buise, Fabienne D. Simonis, Marcus J. Schultz, Jennifer C. Goodson, Troy S. Browne, Leanlove Navarra, Anna Hunt, Robyn A. Hutchison, Mathew B. Bailey, Lynette Newby, Colin McArthur, Michael Kalkoff, Alex Mcleod, Jonathan Casement, DanielleJ Hacking, Finn H. Andersen, Merete S. Dolva, Jon H. Laake, Andreas Barratt-Due, Kim Andre L. Noremark, Eldar Søreide, BritÅ Sjøbø, AnneB Guttormsen, Hector H. LeonYoshido, Ronald Zumaran Aguilar, Fredy A. Montes Oscanoa, Alain U. Alisasis, Joanne B. Robles, Rossini Abbie B. Pasanting-Lim, Beatriz C. Tan, Pawel Andruszkiewicz, Karina Jakubowska, Cristina M. Coxo, António M. Alvarez, Bruno S. Oliveira, Gustavo M. Montanha, Nelson C. Barros, Carlos S. Pereira, António M. Messias, Jorge M. Monteiro, AnaM Araujo, NunoT Catorze, Susan M. Marum, Maria J. Bouw, Rui M. Gomes, Vania A. Brito, Silvia Castro, Joana M. Estilita, Filipa M. Barros, IsabelM Serra, Aurelia M. Martinho, Dana R. Tomescu, Alexandra Marcu, Ovidiu H. Bedreag, Marius Papurica, Dan E. Corneci, Silvius Ioan Negoita, Evgeny Grigoriev, Alexey I. Gritsan, Andrey A. Gazenkampf, Ghaleb Almekhlafi, Mohamad M. Albarrak, Ghanem M. Mustafa, Khalid A. Maghrabi, Nawal Salahuddin, Tharwat M. Aisa, AhmedS AlJabbary, Edgardo Tabhan, YaseenM Arabi, Yaseen M. Arabi, Olivia A. Trinidad, Hasan M. Al Dorzi, Edgardo E. Tabhan, Vesna Bumbasirevic, Bojan Jovanovic, Stefan Bolon, Oliver Smith, Jordi Mancebo, Hernan Aguirre-Bermeo, JuanC Lopez-Delgado, Francisco Esteve, Gemma Rialp, Catalina Forteza, Candelaria De Haro, Antonio Artigas, GuillermoM Albaiceta, Sara De Cima-Iglesias, Leticia Seoane-Quiroga, Alexandra Ceniceros-Barros, AntonioL RuizAguilar, LuisM Claraco-Vega, Juan Alfonso Soler, Maria del CarmenLorente, Cecilia Hermosa, Federico Gordo, Miryam PrietoGonzález, JuanB López-Messa, ManuelP Perez, CesarP Perez, Raquel Montoiro Allue, Ferran RocheCampo, Marcos Ibañez-Santacruz, Susana Temprano, Maria C. Pintado, Raul De Pablo, Pilar Ricart Aroa Gómez, Silvia Rodriguez Ruiz, Silvia Iglesias Moles, M. Teresa Jurado, Alfons Arizmendi, Enrique A. Piacentini, Nieves Franco, Teresa Honrubia, Meisy Perez Cheng, Elena Perez Losada, Javier Blanco, Luis J. Yuste, Cecilia Carbayo-Gorriz, Francisca G. Cazorla-Barranquero, Javier G. Alonso, Rosa S. Alda, Ángela Algaba, Gonzalo Navarro, Enrique Cereijo, Esther Diaz-Rodriguez, Diego Pastor Marcos, Laura Alvarez Montero, Luis Herrera Para, Roberto Jimenez Sanchez, Miguel Angel Blasco Navalpotro, Ricardo Diaz Abad, Raquel Montiel González, Dácil Parrilla Toribio, Alejandro G. Castro, Maria Jose D. Artiga, Oscar Penuelas, Tomas P. Roser, Moreno F. Olga, Elena Gallego Curto, Rocío Manzano Sánchez, Vallverdu P. Imma, Garcia M. Elisabet, Laura Claverias, Monica Magret, Ana M. Pellicer, Lucia L. Rodriguez, Jesús Sánchez-Ballesteros, Ángela González-Salamanca, AntonioG Jimenez, FranciscoP Huerta, Juan Carlos J. Sotillo Diaz, Esther Bermejo Lopez, David D. Llinares Moya, Alec A. Tallet Alfonso, Palazon Sanchez Eugenio Luis, Palazon Sanchez Cesar, Sánchez I. Rafael, CorcolesG Virgilio, NoeliaN Recio, Richard O. Adamsson, Christian C. Rylander, Bernhard Holzgraefe, Lars M Broman, Joanna Wessbergh, Linnea Persson, Fredrik Schiöler, Hans Kedelv, Anna Oscarsson Tibblin, Henrik Appelberg, Lars Hedlund, Johan Helleberg, KarinE Eriksson, Rita Glietsch, Niklas Larsson, Ingela Nygren, SilviaL Nunes, Anna-Karin Morin, Thomas Kander, Anne Adolfsson, HervéO Zender, Corinne Leemann-Refondini, Souheil Elatrous, Slaheddine Bouchoucha, Imed Chouchene, Islem Ouanes, Asma Ben Souissi, Salma Kamoun, Oktay Demirkiran, Mustafa Aker, Emre Erbabacan, Ilkay Ceylan, Nermin Kelebek Girgin, Menekse Ozcelik, Necmettin Ünal, Basak Ceyda Meco, OnatO Akyol, SuleymanS Derman, Barry Kennedy, Ken Parhar, Latha Srinivasa, Lia McNamee, Danny McAuley, Phil Hopkins, Clare Mellis, Vivek Kakar, Dan Hadfield, Andre Vercueil, Kaushik Bhowmick, Sally K. Humphreys, Andrew Ferguson, Raymond Mckee, Ashok S. Raj, Danielle A. Fawkes, Philip Watt, Linda Twohey, Rajeev R. Jha, Matthew Thomas, Alex Morton, Varsha Kadaba, Mark J. Smith, Anil P. Hormis, Santhana G. Kannan, Miriam Namih, Henrik Reschreiter, Julie Camsooksai, Alek Kumar, Szabolcs Rugonfalvi, Christopher Nutt, Orla Oneill, Colette Seasman, Ged Dempsey, ChristopherJ Scott, HelenE Ellis, Stuart Mckechnie, PaulaJ Hutton, Nora N. Di Tomasso, Michela N. Vitale, Ruth O. Griffin, MichaelN Dean, JuliusH Cranshaw, EmmaL Willett, Nicholas Ioannou, Sarah Gillis, Peter Csabi, Rosaleen Macfadyen, Heidi Dawson, PieterD Preez, Alexandra J Williams, Owen Boyd, Laura Ortiz-Ruiz de Gordoa, Jon Bramall, Sophie Symmonds, SimonK Chau, Tim Wenham, Tamas Szakmany, Piroska Toth-Tarsoly, KatieH McCalman, Peter Alexander, Lorraine Stephenson, Thomas Collyer, Rhiannon Chapman, Raphael Cooper, Russell M Allan, Malcolm Sim, David W Wrathall, DonaldA Irvine, Charing Kim S. Zantua, John C. Adams, Andrew J. Burtenshaw, Gareth P. Sellors, Ingeborg D. Welters, Karen E. Williams, Robert J. Hessell, Matthew G. Oldroyd, Ceri E. Battle, Suresh Pillai, Istvan Kajtor, Mageswaran Sivashanmugavel, Sinead C. Okane, Adrian Donnelly, Aniko D. Frigyik, Jon P. Careless, Martin M May, Richard Stewart, T. John Trinder, SamanthaJ Hagan, JadeM Cole, Caroline C. MacFie, AnnaT Dowling, Javier Hurtado, Nicolás Nin, Javier Hurtado, Edgardo Nuñez, Gustavo Pittini, Ruben Rodriguez, María C. Imperio, Cristina Santos, Ana G França, Alejandro Ebeid, Alberto Deicas, Carolina Serra, Aditya Uppalapati, Ghassan Kamel, Valerie M. BannerGoodspeed, Jeremy R. Beitler, Satyanarayana Reddy Mukkera, Shreedhar Kulkarni, John O. Shinn III, Dina Gomaa, Christopher Tainter, Jarone Lee, Tomaz Mesar, DaleJ Yeatts, Jessica Warren, MichaelJ Lanspa, Russel R. Miller, ColinK Grissom, SamuelM Brown, Philippe R. Bauer, Ryan J. Gosselin, Barrett T. Kitch, Jason E. Cohen, Scott H. Beegle, Shazia Choudry, Renaud M. Gueret, Aiman Tulaimat, William Stigler, Hitesh Batra, Nidhi G. Huff, Keith D. Lamb, Trevor W. Oetting, Nicholas M. Mohr, Claine Judy, Shigeki Saito, Fayez M. Kheir, Fayez Kheir, Adam B. Schlichting, Angela Delsing, Daniel R. Crouch, Mary Elmasri, Daniel R. Crouch, Dina Ismail, Kyle R. Dreyer, Thomas C. Blakeman, Dina Gomaa, Rebecca M. Baro, Peter C. Hou, Raghu Seethala, Imo Aisiku, Galen Henderson, Gyorgy Frendl, Sen-Kuang Hou, RobertL Owens, Ashley Schomer

**Affiliations:** 1 Instituto de Investigación Sanitaria del Principado de Asturias, Oviedo, Spain; 2 Unidad de Cuidados Intensivos Cardiológicos, Hospital Universitario Central de Asturias, Avenida del Hospital Universitario s/n, 33011 Oviedo, Spain; 3 Instituto Universitario de Oncología del Principado de Asturias, Universidad de Oviedo, Oviedo, Spain; 4 Centro de Investigación Biomédica en Red (CIBER)-Enfermedades Respiratorias, Madrid, Spain; 5 Department of Medicine and Surgery, University of Milan-Bicocca, Monza, Italy; 6 Department of Emergency and Intensive Care, San Gerardo Hospital, Monza, Italy; 7 Service de Médecine Intensive-Réanimation, AP-HP, Hôpital de Bicêtre, DMU 4 CORREVE Maladies du Cœur et des Vaisseaux, FHU Sepsis, Groupe de Recherche Clinique CARMAS, Le Kremlin-Bicêtre, France; 8 Université Paris-Saclay, UVSQ, Inserm U1018, Equipe d’Epidémiologie Respiratoire Intégrative, CESP, 94807 Villejuif, France; 9 Department of Medicine, University Health Network and Mount Sinai Hospital, Toronto, ON Canada; 10 Interdepartmental Division of Critical Care Medicine and Institute of Health Policy, Management and Evaluation, University of Toronto, Toronto, ON Canada; 11 Department of Anesthesia, Critical Care and Emergency‚ Fondazione IRCCS Ca’ Granda Ospedale Maggiore Policlinico, Milan, Italy; 12 Department of Anaesthesia and Intensive Care Medicine, Galway University Hospitals, Galway, Ireland; 13 School of Medicine, Regenerative Medicine Institute at CÚRAM Centre for Research in Medical Devices, University of Galway, Galway, Ireland

**Keywords:** Mechanical ventilation, Cardiogenic pulmonary edema, Ventilator-induced lung injury, Driving pressure

## Abstract

**Background:**

Patients with acute respiratory failure caused by cardiogenic pulmonary edema (CPE) may require mechanical ventilation that can cause further lung damage. Our aim was to determine the impact of ventilatory settings on CPE mortality.

**Methods:**

Patients from the LUNG SAFE cohort, a multicenter prospective cohort study of patients undergoing mechanical ventilation, were studied. Relationships between ventilatory parameters and outcomes (ICU discharge/hospital mortality) were assessed using latent mixture analysis and a marginal structural model.

**Results:**

From 4499 patients, 391 meeting CPE criteria (median age 70 [interquartile range 59–78], 40% female) were included. ICU and hospital mortality were 34% and 40%, respectively. ICU survivors were younger (67 [57–77] *vs* 74 [64–80] years, *p* < 0.001) and had lower driving (12 [8–16] *vs* 15 [11–17] cmH_2_O, *p* < 0.001), plateau (20 [15–23] *vs* 22 [19–26] cmH_2_O, *p* < 0.001) and peak (21 [17–27] *vs* 26 [20–32] cmH_2_O, *p* < 0.001) pressures. Latent mixture analysis of patients receiving invasive mechanical ventilation on ICU day 1 revealed a subgroup ventilated with high pressures with lower probability of being discharged alive from the ICU (hazard ratio [HR] 0.79 [95% confidence interval 0.60–1.05], *p* = 0.103) and increased hospital mortality (HR 1.65 [1.16–2.36], *p* = 0.005). In a marginal structural model, driving pressures in the first week (HR 1.12 [1.06–1.18], *p* < 0.001) and tidal volume after day 7 (HR 0.69 [0.52–0.93], *p* = 0.015) were related to survival.

**Conclusions:**

Higher airway pressures in invasively ventilated patients with CPE are related to mortality. These patients may be exposed to an increased risk of ventilator-induced lung injury.

*Trial registration* Clinicaltrials.gov NCT02010073

**Supplementary Information:**

The online version contains supplementary material available at 10.1186/s40560-022-00648-x.

## Background

Lung edema causes respiratory failure due to impairment in gas exchange and lung mechanics. In life-threatening cases, mechanical ventilation aims to maintain gas exchange until edema is resolved. However, high airway pressures may promote ventilator-induced lung injury (VILI) [1]. In patients with the acute respiratory distress syndrome (ARDS), ventilatory strategies aimed to attenuate VILI (by decreasing tidal volumes and driving pressures) have improved survival [2].

In patients with cardiogenic pulmonary edema (CPE), airspaces are flooded due to capillary congestion. Although CPE lacks an inflammatory component, its heterogeneous distribution, impaired gas exchange and respiratory mechanics and high mortality rates are shared features with ARDS [3, 4]. Mortality rates in cardiogenic pulmonary edema and cardiogenic shock remain high, with only minor improvements in the last years [5, 6]. Patients with CPE that need mechanical ventilation constitute a subgroup with a significant mortality [7–9], and the incidence of respiratory failure within patients admitted to cardiac intensive care units may be increasing [10, 11].

In spite of the impact of mechanical ventilation on mortality rates in patients with CPE, there is no clear evidence on the optimal ventilatory settings. Although experience with mechanical ventilation is related to better outcomes in cardiac ICUs [12], patients with CPE have traditionally been excluded from trials on mechanical ventilation and the risk of VILI has not been systematically addressed [13]. Two previous retrospective reports have associated high tidal volumes or driving pressures with mortality in patients with CPE [14, 15].

We hypothesized that patients with CPE are susceptible to VILI and their outcomes sensitive to ventilatory strategies. To test this hypothesis, patients with isolated CPE included in the LUNG SAFE study [16] were selected to study the relationships between mechanical ventilation and clinical outcomes in this population.

## Methods

### Study design

LUNG SAFE (clinicaltrials.gov NCT02010073) was a multicenter (459 ICUs from 50 countries), prospective cohort study that enrolled 4499 patients with hypoxemic respiratory failure [16]. Patients aged < 16 years or not willing to participate were excluded. Only patients with respiratory failure from isolated cardiac origin were included in this sub-study. All participating ICUs obtained ethics committee approval and obtained either patient consent or ethics committee waiver of consent (due to the observational nature of the study).

### Data collection

Clinical data, including cause of respiratory failure, concomitant diseases and ICU and hospital outcomes were collected. Day 1 was defined as the first day meeting acute hypoxemic respiratory failure (defined as a PaO_2_/FiO_2_ ratio below 300, appearance of parenchymal abnormalities in a chest X-ray and need for ventilatory support, either invasive or non-invasive, with an airway pressure equal or above 5 cmH_2_O). Daily data, including ventilatory settings, gas exchange, Sequential Organ Failure Assessment (SOFA) score and concomitant respiratory therapies, were collected on days 1, 2, 3, 5, 7, 10, 14, 21, and 28 at the same hour (usually 10 AM) by the research team. Plateau pressures in patients under pressure-controlled or -assisted modes were considered equal to peak pressures.

### Patient selection

Presence of heart failure at Day 1 was identified according to the responsible researcher. Methods used to confirm/discard cardiac dysfunction were collected.

### Follow-up and outcomes

Patients were followed up to hospital discharge. Primary and secondary outcomes were ICU discharge alive and spontaneously breathing, and hospital mortality, respectively. Prolonged mechanical ventilation was defined as need for mechanical ventilation for more than 10 days (the 75th percentile of length of ventilation).

### Statistical analysis

Data are expressed as median (interquartile range) or count (percentage). Univariable comparisons were analyzed using Wilcoxon or Chi-square tests. Differences over time between groups were assessed using a repeated measurements analysis of the variance. Correlations between ventilatory parameters and the hemodynamic component of SOFA score were assessed using Spearman’s coefficients.

Patients were classified using a latent mixture analysis in two mutually exclusive and exhaustive classes using peak, plateau and driving pressures, positive end-expiratory pressure (PEEP), tidal volume (adjusted by predicted body weight), respiratory system compliance, PaO_2_/FiO_2_ and respiratory rate on day 1. Differences in ICU survival and hospital mortality between these two classes were analyzed using a competing events model, with ICU discharge (alive and spontaneously breathing) and death as terminal events.

To identify factors related to ICU survival, inverse probability of treatment weights were calculated for driving pressure, so that each observation is weighted by the inverse of the probability of the exposure, given the observed value of other confounders [tidal volume, PaO_2_/FiO_2_ ratio, PaCO_2_, PEEP and history of chronic obstructive pulmonary disease (COPD)]. These weights were introduced in a marginal structural model including all available data points for a given patient, and a weighted Cox regression used to calculate the hazard ratio (HR) and its 95% confidence interval for each variable. Variables included in this Cox model were age, sex, chronic renal failure, chronic heart failure, COPD, PaO_2_/FiO_2_, PaCO_2_, class assigned by the latent mixture model, tidal volume (adjusted by predicted body weight), driving pressure and PEEP. By adding a time stratum, different HRs were computed for ventilatory parameters (driving pressure, tidal volume and PEEP) before and after day 7. All analyses were performed with R 4.1.0, using the packages survival [17], ipw [18], depmixS4 [19], ggplot2 [20] and ggfortify [21].

## Results

**Fig. 1:**
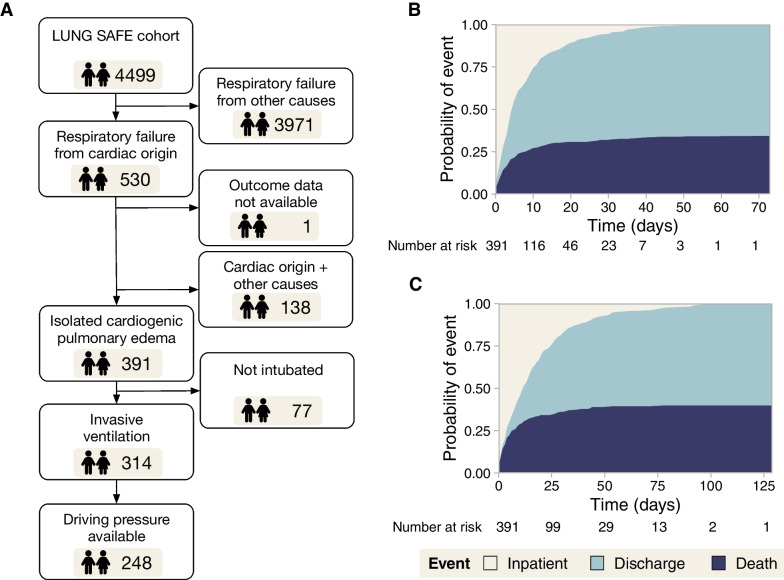
**A** Patient flowchart. **B** Intensive care unit (ICU) survival and mortality. **C** Hospital survival and mortality

From the 4499 patients with acute hypoxemic respiratory failure included in LUNG SAFE, 530 were classified as having a component of heart failure contributing to their respiratory failure. One patient was excluded due to absence of outcome data. In 138 patients, another cause of respiratory failure was registered, leaving 391 patients (age 70 [59–78], 40% female) with isolated CPE (Fig. 1A). The diagnosis of cardiac failure was supported by echocardiography (314 cases), pulmonary artery catheter (56 cases) and other techniques (49 cases). In 46 cases, diagnosis was based on clinical data only.

Median ICU and hospital stay were 5 (2–11) and 14 (5–27) days, respectively. ICU mortality was 34%, and increased up to 40% at hospital discharge (Fig. 1B and C, respectively). ICU survivors were younger and received non-invasive ventilation more frequently at admission. Non-survivors showed significantly higher airway pressures on day 1 (Table 1).

**Table 1:** Main clinical variables in the overall study cohort, comparing patients who survive their ICU stay and those who do not

	Overall (*N* = 391)	Alive (*N* = 257)	Dead (*N* = 134)	*p*-value
Gender				0.286
Female	156 (40%)	97 (38%)	59 (44%)	
Male	236 (60%)	160 (62%)	75 (56%)	
Age (year)	70 (59–78)	67 (57–77)	74 (64–80)	< 0.001
Predicted body weight (kg)	60 (52–67)	60 (52–67)	59 (52–64)	0.241
Chronic heart failure	165 (42%)	107 (42%)	58 (43%)	0.862
Comorbidities				
Diabetes	120 (31%)	80 (31%)	40 (30%)	0.866
Chronic kidney failure	77 (20%)	51 (20%)	26 (19%)	1
Chronic liver failure	8 (2%)	3 (1%)	5 (4%)	0.188
Solid neoplasm	12 (3%)	6 (2%)	6 (4.5%)	0.395
Hematological neoplasm	6 (1.5%)	4 (1.5%)	2 (1.5%)	1
Chronic immunosuppression	8 (2%)	6 (2%)	2 (1.5%)	0.852
COPD	56 (14%)	31 (12%)	25 (19%)	0.11
Home ventilation	6 (1.5%)	2 (1%)	4 (3%)	0.213
*Day 1*				
SOFA score	9 (7–12)	9 (6–11)	11 (9–14)	< 0.001
Hemodynamic SOFA score^a^				< 0.001
MAP ≥ 70 mmHg	114 (31%)	94 (39%)	20 (16%)	
MAP < 70 mmHg	40 (11%)	22 (9%)	18 (14%)	
Dopamine ≤ 5 μg/kg/min or dobutamine	25 (7%)	16 (7%)	9 (7%)	
Dopamine 5–15 μg/kg/min or norepinephrine ≤ 0.1 μg/kg/min or epinephrin ≤ 0.1 μg/kg/min	61 (16%)	41 (17%)	20 (16%)	
Dopamine > 15 μg/kg/min or norepinephrine > 0.1 μg/kg/min or epinephrin > 0.1 μg/kg/min	127 (35%)	68 (28%)	59 (47%)	
Arterial pH	7.35 (7.27–7.43)	7.38 (7.29–7.43)	7.32 (7.24–7.40)	< 0.001
PaO_2_/FiO_2_	172 (116–231)	176 (125–232)	164 (109–231)	0.165
PaCO_2_(mmHg)	40 (34–48)	40 (35–48)	40 (34–48)	0.911
Ventilation				0.001
None/oxygen therapy	10 (2.5%)	5 (2%)	5 (4%)	
Non-invasive ventilation	67 (17%)	57 (22%)	10 (7%)	
Invasive ventilation	314 (80.5%)	195 (76%)	119 (89%)	
Tidal volume (ml/kg PBW)	8.2 (7.2–9.4)	8.3 (7.3–9.4)	8.1 (7.0–9.3)	0.237
PEEP (cmH_2_O)	7 (5–8)	7 (5–8)	6 (5–8)	0.197
Peak pressure (cmH_2_O)	22 (18–28)	21 (17–27)	26 (20–32)	< 0.001
Plateau pressure (cmH_2_O)	20 (16–24)	20 (15–23)	22 (19–26)	< 0.001
Driving pressure (cmH_2_O)	13 (9–16)	12 (8–16)	15 (11–17)	< 0.001
Respiratory rate (breaths/min)	18 (15–22)	18 (15–22)	19 (15–24)	0.196
*ICU evolution*				
ARDS development				
Invasive mechanical ventilation	327 (84%)	204 (79%)	123 (92%)	0.002
Venoarterial ECMO	18 (5%)	6 (2%)	12 (9%)	0.007
Renal replacement therapy	74 (19%)	38 (15%)	36 (27%)	0.006
Length of mechanical ventilation (days)	4 (2–10)	5 (3–10)	4 (2–9)	0.208

Values represent median (interquartile range) or count (percentage). *p*-values were obtained using Wilcoxon or Chi-square tests (for quantitative and qualitative data, respectively). In variables with multiple mutually exclusive categories per group (i.e., hemodynamics or ventilation), a Chi-square test including all the categories was performed. Tidal volume, airway pressures and respiratory rate are reported only for patients on invasive ventilation. PBW: predicted body weight, COPD: chronic obstructive pulmonary disease, PEEP: positive end-expiratory pressure

^a^SOFA score at day 1 was available for 367 patients

### Non-invasive ventilation

Sixty-seven patients received non-invasive ventilation as first-line ventilatory therapy. There were no differences in age, sex or previous diseases between these patients and those who received invasive ventilation (Additional file 1: Table S1). Patients treated with non-invasive ventilation had lower SOFA scores, less hemodynamic impairment and lower driving pressure with the same levels of PEEP (Additional file 1: Table S1). Only 9 out of these 67 patients (13%) required invasive ventilation. ICU mortality was lower in patients who received non-invasive ventilation (15% vs 39%, *p* < 0.001, Additional file 1: Table S1).

### Clustering by ventilatory settings

**Fig. 2:**
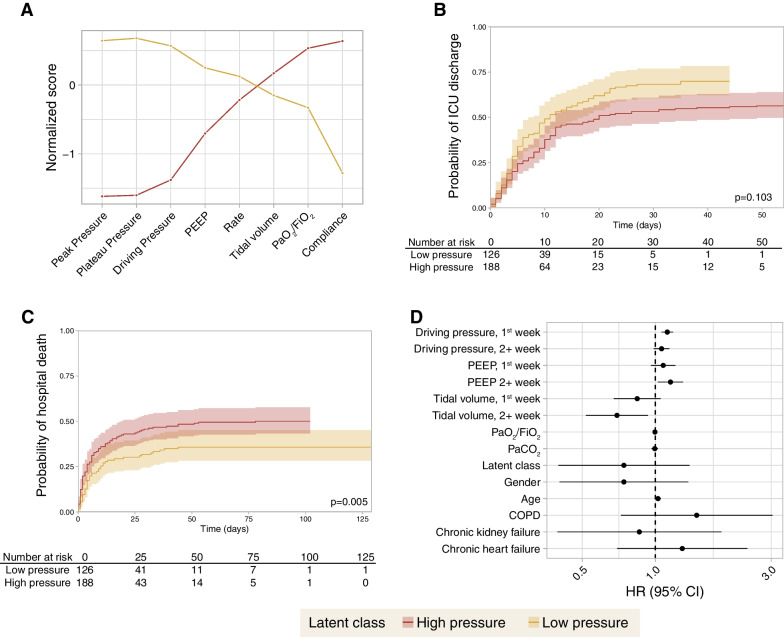
**A** Profile plot of two patient classes identified according to several ventilatory variables at first day of invasive mechanical ventilation using a latent mixture analysis. Values show means in each variable used for classification for each class (after normalization, Z-scores). The largest differences are observed in airway pressures, whereas there are no differences in respiratory rate. **B** Discharge from the intensive care unit (ICU) alive and spontaneously breathing for each patient class. **C** Hospital mortality for each patient class. **D** Hazard ratios for mortality obtained from a marginal structural model addressing the time-dependent changes in each variable

A latent mixture analysis identified two classes in invasively ventilated patients (n = 314), based on respiratory parameters at study inclusion (“high pressure” [n = 188] and “low pressure” [n = 126], Fig. 2A). Class assignment probability was below 0.7 in only 39 out of 314 patients. Fitting the model with three classes increased this number of patients up to 156. Differences between groups are shown in Table 2. Patients in the high-pressure class showed a lower probability of being discharged alive from the ICU (HR 0.79 [0.60 – 1.05], Fig. 2B), and increased hospital mortality (HR 1.65 [1.16—2.36], Fig. 2C).

**Table 2:** Comparison of clinical data from patients assigned to high- or low-pressure classes by the latent mixture analysis

	High pressure (*N* = 188)	Low pressure (*N* = 126)	*p*.value
Gender			0.338
Female	77	44	
Male	111	82	
Age (year)	69 (61–77)	79 (59–77)	0.773
Predicted body weight (kg)	60.2 (52.4–66.3)	61 (53–67.1)	0.661
Chronic heart failure	74 (39%)	53 (42%)	0.718
Comorbidities			
Diabetes	58 (31%)	30 (24%)	0.217
Chronic kidney failure	43 (23%)	17 (13%)	0.054
Chronic liver failure	5 (3%)	2 (2%)	0.81
Solid neoplasm	4 (2%)	7 (6%)	0.191
Hematological neoplasm	2 (1%)	3 (2%)	0.65
Chronic immunosuppression	5 (3%)	1 (1%)	0.704
COPD	31 (16%)	15 (12%)	0.335
Home ventilation	5 (3%)	0 (0%)	0.166
*Day 1*			
SOFA score	11 (9–13)	9 (7–12)	0.002
Hemodynamic SOFA score^a^			< 0.001
MAP ≥ 70 mmHg	52 (23%)	62 (46%)	
MAP < 70 mmHg	23 (10%)	17 (13%)	
Dopamine ≤ 5 μg/kg/min or dobutamine	15 (6%)	10 (7%)	
Dopamine 5–15 μg/kg/min or norepinephrine ≤ 0.1 μg/kg/min or epinephrin ≤ 0.1 μg/kg/min	46 (20%)	15 (11%)	
Dopamine > 15 μg/kg/min or norepinephrine > 0.1 μg/kg/min or epinephrin > 0.1 μg/kg/min	95 (41%)	32 (23%)	
Arterial pH	7.33 (7.24–7.41)	7.38 (7.31–7.43)	0.006
PaO_2_/FiO_2_	148 (100–189)	219 (163–247)	< 0.001
PaCO_2_(mmHg)	40 (35–48)	40 (34–47)	0.726
Tidal volume (ml/kg PBW)	8.1 (7.2–9.0)	8.7 (7.2–9.8)	0.014
PEEP (cmH_2_O)	8 (5–10)	6 (5–7)	< 0.001
Peak pressure (cmH_2_O)	28 (24–34)	19 (16–21)	< 0.001
Plateau pressure (cmH_2_O)	24 (21–28)	17 (14–19)	< 0.001
Driving pressure (cmH_2_O)	16 (14–19)	10 (7–12)	< 0.001
Respiratory rate (breaths/min)	19 (15–22)	17 (14–20)	0.004
Respiratory system compliance (ml/cmH_2_O)	30 (25–37)	54 (43–72)	< 0.001
*ICU evolution*			
Death in ICU	81 (43%)	38 (30%)	0.028
Causes of death			0.224
Cardiovascular failure	60 (74%)	26 (68%)	
Neurologic failure	11 (14%)	7 (18%)	
Respiratory failure	4 (5%)	3 (8%)	
Other	6 (7%)	2 (6%)	
Length of mechanical ventilation (days)	4 (2–10)	5 (2–10)	0.828
Ventilator-free days	4 (0–24)	19 (0–26)	0.006
*Hospital evolution*			
Death in hospital	93 (49%)	45 (36%)	0.022

Values represent median (interquartile range) or count (percentage). p-values were obtained using Wilcoxon or Chi-square tests (for quantitative and qualitative data, respectively). In variables with multiple mutually exclusive categories per group (i.e., hemodynamics), a Chi-square test including all the categories was performed

^a^ SOFA score was available for 166 patients

**Fig. 3:**
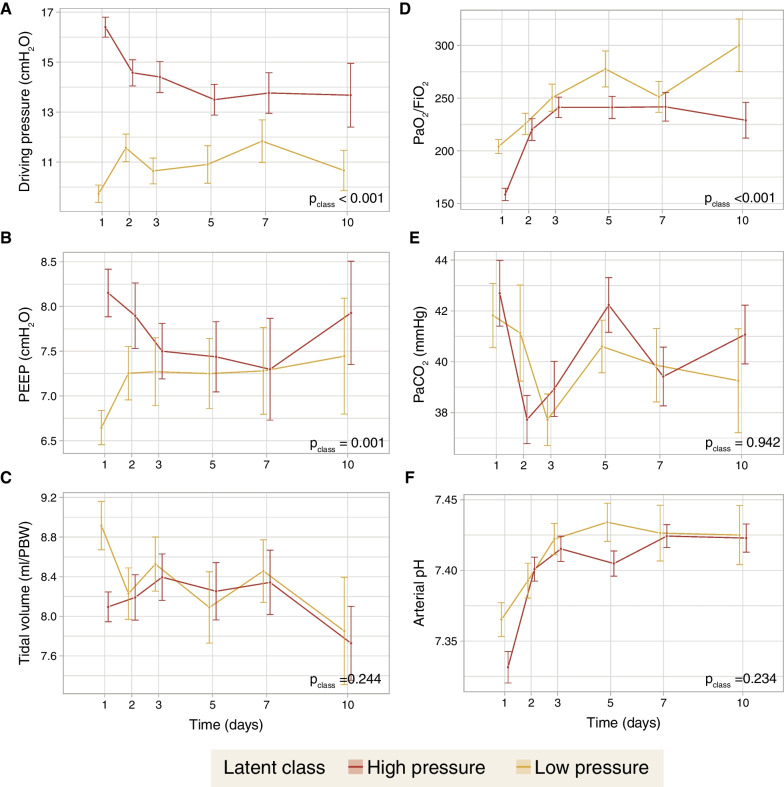
Time course of ventilatory settings and gas exchange over the first 10 days, according to the previously identified latent classes. **A** Driving pressures. **B** Positive end-expiratory pressure (PEEP). **C** Tidal volumes, expressed as milliliters per predicted body weight (PBW). **D** PaO_2_/FiO_2_ ratio. **E** PaCO_2_. **F** Arterial pH. Values were compared between classes using a repeated measurements analysis of the variance (ANOVA)

The differences in ventilatory parameters over time between classes were assessed. Whereas differences in driving pressure (Fig. 3A) persisted over the first 10 days, differences in PEEP (Fig. 3B) were restricted to days 1–3. There were no differences in tidal volumes (Fig. 3C). Regarding gas exchange, there were significant differences in PaO_2_/FiO_2_ (Fig. 3D), but not in PaCO_2_ (Fig. 3E) or arterial pH (Fig. 3F).

### Prolonged ventilation

Invasive mechanical ventilation was needed in 327 patients, with a median length of 4 (2 – 10) days. There were 66 patients with prolonged ventilation (more than the 75^th^ percentile of length of ventilation). When compared to those patients with a shorter duration of mechanical ventilation (Additional file 1: Table S2), there were no significant differences in any variable collected at day 1 other than tidal volume, which was lower in patients with prolonged ventilation. The rates of mechanical circulatory support or renal replacement therapy were also similar between groups. However, development of acute respiratory distress syndrome during the ICU stay was more common in patients with prolonged ventilation.

### Relationships between ventilatory settings and hemodynamics

**Fig. 4:**
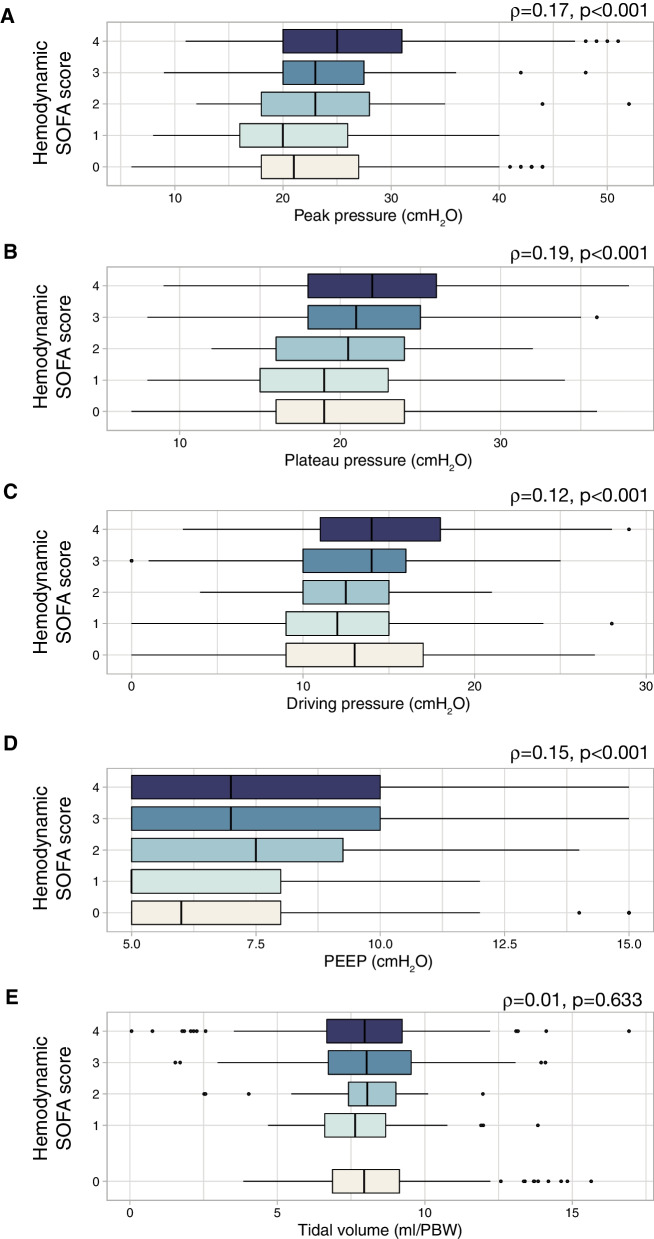
Distribution of values of ventilatory settings (**A** Peak inspiratory pressure; **B** Plateau pressure; **C** Driving pressure; **D** Positive end-expiratory pressure [PEEP]; **E** Tidal volume) according to the hemodynamic item of the SOFA score (0: mean arterial pressure ≥ 70 mmHg; 1: mean arterial pressure < 70 mmHg, 2: dopamine ≤ 5 μg/kg/min or dobutamine; 3: dopamine 5–15 μg/kg/min or norepinephrine ≤ 0.1 μg/kg/min or epinephrin ≤ 0.1 μg/kg/min; 4: dopamine > 15 μg/kg/min or norepinephrine > 0.1 μg/kg/min or epinephrin > 0.1 μg/kg/min). Spearman’s coefficients (*ρ*) were calculated to assess the correlation between these parameters and the score

Distribution of ventilatory settings along the different levels of hemodynamic impairment (measured using the hemodynamic item of the SOFA score) was explored. Peak pressure (Fig. 4A), plateau pressure (Fig. 4B), driving pressure (Fig. 4C) and PEEP (Fig. 4D) were positively correlated with the hemodynamic SOFA score, showing weak, but significant correlations. However, no correlation was observed for tidal volumes (Fig. 4E).

### Role of driving pressure on survival

We fitted a marginal structural model in 248 patients with invasive ventilation and plateau pressure data. High driving pressures during the first ICU week were related to a significant increase in mortality, whereas high tidal volumes after day 7 showed the opposite (Fig. 2D and Additional file 1: Table S3). Several sensitivity analyses were performed: restricting the analysis to those patients on controlled invasive ventilation on day 1 (Additional file 1: Table S4), after exclusion of imputed plateau pressures (Additional file 1: Table S5), after excluding patients with only a clinical diagnosis of CPE (Additional file 1: Table S6), using dynamic driving pressures (measured as peak pressure minus PEEP, Additional file 1: Table S7), or inclusion of patients on non-invasive ventilation on day 1 (Additional file 1: Table S8), did not substantially modify these findings (Additional file 1: Fig. S1).

## Discussion

In this sub-study of the LUNG SAFE cohort, patients with isolated cardiogenic respiratory failure showed 40% of hospital mortality, similar to that observed for ARDS patients and recent series of patients with cardiogenic shock requiring mechanical ventilation [8, 22, 23]. Our results show that high driving pressures during the first week of ventilation were associated to a significantly increased mortality, supporting the impact of mechanical ventilation on the outcomes of patients with CPE.

Alveolar edema results in reduced functional residual capacity and compliance, and may promote lung injury when high tidal volumes are applied. Alveolar flooding caused by hydrostatic, non-inflammatory mechanisms, may replicate a “baby lung” effect in patients with CPE [3]. This reduction in the airspaces available for ventilation increases the susceptibility to VILI by diverting the bulk of tidal volume towards aerated areas, causing local overdistension and increased pressures. The increased cell stretch may trigger lung inflammation, causing or perpetuating lung injury and systemic inflammation. Although the importance of VILI and the optimal ventilatory settings in patients without pre-existing inflammation remains unclear^.^[24], there is increasing evidence that lung inflammation may play a role in the pathogenesis of CPE and cardiogenic shock [25]. Interestingly, new onset of ARDS was the only variable related to prolonged ventilation in this cohort, highlighting the importance of a second hit on the outcome. However, the specific contribution of VILI to perpetuate cardiovascular failure, which is the most common cause of death in our cohort, is unknown.

Non-invasive ventilation can help to ensure gas exchange while avoiding intubation, and its use has yielded better outcomes in CPE [26, 27]. Our study corroborates these lower mortality rates in patients receiving non-invasive ventilation. It is unclear if these patients are exposed to an increased risk of ventilator-induced lung injury, although it has been proposed that large spontaneous inspiratory efforts may cause damage (termed patient self-inflicted lung injury) [28]. Although airway pressures during non-invasive ventilation were lower, our available data cannot discard an increased contribution of spontaneous breathing to these pressures, thus increasing transpulmonary pressures.

There is substantial debate on how different ventilatory settings are related to VILI. As previously described, tidal volume may promote regional overdistension. The use of reduced tidal volumes (6 ml/kg) decreased mortality in ARDS. Tidal volumes used in this CPE cohort are substantially higher than this value, and a threshold of 9 ml/kg has been correlated with a worse outcome [14]. From a pathogenetic point of view, tidal volume is a global measurement and local phenomena are driven by changes in pressure, which is sensed locally by lung cells. Hence, driving pressure, rather than tidal volume alone, has been proposed as a better marker of regional lung strain with better correlation to mortality than tidal volume in ARDS [29]. Respiratory system compliance, as a marker of the amount of lung available for ventilation, emerges then as a relevant biomarker to identify the risk of VILI. Our results, using a weighted marginal structural model that isolates the effects of driving pressures from other confounders, support the association between driving pressures and mortality in patients with CPE. On the other hand, PEEP, which is a major determinant of lung recruitment, may have multifaceted effects on VILI, as increasing end-expiratory volume may promote the recruitment of collapsed or flooded alveoli for ventilation, but also cause overdistension of previously aerated areas [30].

Heart–lung interactions are a major concern in mechanically ventilated patients [31]. Our data show progressively increased airway pressures according to the severity of the hemodynamic impairment. The increase in peak, plateau and driving pressures, with no change in tidal volume, can be explained by a progressive decrease in lung compliance. These results raise the hypothesis that clinicians set tidal volume to ensure ventilation, and the obtained pressures are the consequence of the magnitude of lung edema and its impact on respiratory mechanics. Regarding PEEP, that is usually set at lower levels in these patients due to its potential hemodynamic effects [13], no conclusion can be extracted.

This study has some limitations that must be discussed. Available data do not include information on previous cardiac diseases or triggering events. Similarly, the database did not differentiate between patients with preserved or reduced ejection fraction or other heart failure phenotypes [32, 33] although it has been reported that respiratory support is related to worse outcomes in both groups [34]. Therefore, we cannot discard differences in the observed effects of mechanical ventilation on the outcome among CPE phenotypes. Instead, the hemodynamic item of SOFA score was used to categorize the circulatory status at admission. It has been shown that SOFA score has a good prognostic value in patients with heart failure, independently of the baseline ejection fraction [35, 36]. In addition, the observational design allows only for associative conclusions, although the use of inverse probability of treatment weights in a marginal structural model increases the strength of this association by standardizing baseline risks [37]. Finally, plateau pressures were available in 75% of all patients. When a pressure control mode or non-invasive ventilation was registered, inspiratory pressure was considered as plateau pressure. However, excluding these patients from the analysis yielded the same results.

## Conclusions

Our findings highlight the impact of mechanical ventilation in patients with CPE. Although the observational nature of this study prevents any causality relationship to be inferred, our results show that ventilatory variables could be used as a marker of severity and suggest that patients with CPE may be susceptible to VILI. Clinical trials of low tidal volume in CPE should test this hypothesis.

## Supplementary Information

**Additional file 1: Table S1. **Differences between patients who received non-invasive and invasive ventilation as first-line respiratory support.** Table S2. **Differences between patients with not prolonged and prolonged invasive mechanical ventilation (more than 10 days). ** Table S3. **Hazard ratio for mortality of each variable included in the marginal structural model (n=248).** Table S4.** Hazard ratio for mortality of each variable included in a marginal structural model including only patients under controlled mechanical ventilation on ICU day 1 (N=209). ** Table S5.** Hazard ratio for mortality of each variable included in a marginal structural model excluding patients in which plateau pressures were imputed (n= 167 patients). ** Table S6.** Hazard ratio for mortality of each variable included in a marginal structural model excluding patients in which diagnosis of CPE is based only on clinical observations and not supported by any diagnostic technique (n= 229 patients). ** Table S7. **Hazard ratio for mortality of each variable included in a marginal structural model using dynamic driving pressures (peak inspiratory pressure minus PEEP) (n=287). ** Table S8.** Hazard ratio for mortality of each variable included in a marginal structural model including patients without invasive mechanical ventilation at ICU admission (n=295). ** Figure S1.** Hazard ratios (HRs) and 95% confidence intervals (95% CI) of driving pressure obtained in different sensitivity analyses described in Tables S3–S7. CPE: Cardiogenic pulmonary edema.

## Data Availability

Data access policy for LUNG SAFE study is available to researchers at https://www.esicm.org/trials-group-2-lung-safe/.

## Abbreviations

ARDS: Acute respiratory distress syndrome
COPD: Chronic obstructive pulmonary disease
CPE: Cardiogenic pulmonary edema
HR: Hazard ratio
PEEP: Positive end-expiratory pressure
SOFA: Sequential organ failure assessment
VILI: Ventilator-induced lung injury
