# Supplementary material for: Mechanical ventilation in patients with cardiogenic pulmonary edema: a sub-analysis of the LUNG SAFE study
Source: J Intensive Care. 2022 Dec 25;10:55. doi: 10.1186/s40560-022-00648-x (PMC9791731; doi:10.1186/s40560-022-00648-x)

Additional file 1

Mechanical ventilation in patients with cardiogenic pulmonary edema: a sub-analysis of the LUNG SAFE study

Amado-Rodríguez L, Rodríguez-Garcia R, Bellani G et al.

Contents

List of LUNG SAFE investigators.

Table S1. Differences between patients who received non-invasive and invasive ventilation as first-line respiratory support.

Table S2. Differences between patients with not prolonged and prolonged invasive mechanical ventilation (more than 10 days).

Table S3. Hazard ratio for mortality of each variable included in the marginal structural model (n=248).

Table S4. Hazard ratio for mortality of each variable included in a marginal structural model including only patients under controlled mechanical ventilation on ICU day 1 (N=209).

Table S5. Hazard ratio for mortality of each variable included in a marginal structural model excluding patients in which plateau pressures were imputated (n= 167 patients).

Table S6. Hazard ratio for mortality of each variable included in a marginal structural model excluding patients in which diagnosis of CPE is based only on clinical observations and not supported by any diagnostic technique (n= 229 patients).

Table S7. Hazard ratio for mortality of each variable included in a
marginal structural model using dynamic driving pressures (peak inspiratory pressure minus PEEP) (n=287).

Table S8. Hazard ratio for mortality of each variable included in a
marginal structural model including patients without invasive mechanical ventilation at ICU admission (n=295).

Figure S1. Hazard ratios (HRs) and 95% confidence intervals (95% CI)
of driving pressure obtained in different sensitivity analyses described in supplementary tables 3-7. CPE: Cardiogenic pulmonary edema.

LUNG SAFE investigators

LUNG SAFE Steering Committee: Antonio Pesenti, John G. Laffey, Laurent Brochard, Andres Esteban, Luciano Gattinoni, Frank van Haren, Anders Larsson, Daniel F. McAuley, Marco Ranieri, Gordon Rubenfeld, B. Taylor Thompson, Hermann Wrigge and Arthur S. Slutsky. LUNG SAFE Executive Committee: John G. Laffey, Giacomo Bellani, Tài Pham and Eddy Fan. LUNG SAFE national coordinators: Argentina: Fernando Rios; Australia/New Zealand: Frank Van Haren; Belgium: Thierry Sottiaux and Pieter Depuydt; Bolivia: Fredy S. Lora; Brazil: Luciano Cesar Azevedo; Canada: Eddy Fan; Chile: Guillermo Bugedo; China: Haibo Qiu; Colombia: Marcos Gonzalez; Costa Rica: Juan Silesky; Czech Republic: Vladimir Cerny; Denmark: Jonas Nielsen; Ecuador: Manuel Jibaja; France: Tài Pham; Germany: Hermann Wrigge; Greece: Dimitrios Matamis; Guatemala: Jorge Luis Ranero; India: Pravin Amin; Iran: S.M. Hashemian; Ireland: Kevin Clarkson; Italy: Giacomo Bellani; Japan: Kiyoyasu Kurahashi; Mexico: Asisclo Villagomez; Morocco: Amine Ali Zeggwagh; The Netherlands: Leo M. Heunks; Norway: Jon Henrik Laake; Philippines: Jose Emmanuel Palo; Portugal: Antero do Vale Fernandes; Romania: Dorel Sandesc; Saudi Arabia: Yaasen Arabi; Serbia: Vesna Bumbasierevic; Spain: Nicolas Nin and Jose A. Lorente; Sweden: Anders Larsson; Switzerland: Lise Piquilloud; Tunisia: Fekri Abroug; UK: Daniel F. McAuley and Lia McNamee; Uruguay: Javier Hurtado; USA: Ed Bajwa; Venezuela: Gabriel Démpaire. LUNG SAFE site investigators (by country): Albania: UHC Mother Theresa, Tirana: Hektor Sula and Lordian Nunci; University Hospital Shefqet Ndroqi, Tirana: Alma Cani. Argentina: Clinica de Especialidades, Villa Maria: Alan Zazu; Hospital Julio C. Perrando, Resistencia: Christian Dellera and Carolina S. Insaurralde; Sanatorio Las Lomas, San Isidro, Buenos Aires: Risso V. Alejandro; Sanatorio de La Trinidad San Isidro, San Isidro: Julio Daldin and Mauricio Vinzio; Hospital Español de Mendoza, Godoy Cruz-Mendoza: Ruben O. Fernandez; Hospital del Centenario, Rosario: Luis P. Cardonnet and Lisandro R. Bettini; San Antonio, Gualeguay, Entre Rios: Mariano Carboni Bisso and Emilio M. Osman; Cemic, Buenos Aires: Mariano G. Setten and Pablo Lovazzano; Hospital Universitrario Austral, Pilar: Javier Alvarez and Veronica Villar; Hospital Por + Salud, Pami Dr Cesar Milstein, Buenos Aires: Norberto C. Pozo and Nicolas Grubissich; Sanatorio Anchorena, Buenos Aires: Gustavo A. Plotnikow and Daniela N. Vasquez; Sanatorio de La Trinidad Mitre, Buenos Aires: Santiago Ilutovich and Norberto Tiribelli; Hospital Luis agomaggiore, Mendoza: Ariel Chena and Carlos A. Pellegrini; Hospital Interzonal General de Agudos, San Martín, La Plata: María G. Saenz and Elisa Estenssoro; Hospital Misericordia, Cordoba: Matias Brizuela and Hernan Gianinetto; Sanatorio Juncal, Temperley: Pablo E. Gomez and Valeria I.

Cerrato; Hospital D.F. Santojanni, Buenos Aires: Marco G. Bezzi and Silvina A. Borello; Hospital Alejandro Posadas, Buenos Aires: Flavia A. Loiacono and Adriana M. Fernandez. Australia: St Vincent's Hospital, Sydney: Serena Knowles and Claire Reynolds; St George Public Hospital, Kogarah: Deborah M. Inskip and Jennene J. Miller; Westmead Hospital, Westmead: Jing Kong and Christina Whitehead; Flinders Medical Center, Bedford Park: Shailesh Bihari; John Hunter Hospital, Newcastle: Aylin Seven and Amanda Krstevski; Canberra Hospital, Garran: Helen J. Rodgers and Rebecca T. Millar; Calvary Mater Newcastle, Waratah: Toni E. McKenna and Irene M. Bailey; Cabrini Hospital, Melbourne: Gabrielle C. Hanlon; Liverpool Hospital, Liverpool: Anders Aneman and Joan M. Lynch; Coffs Harbour Health Campus, Coffs Harbour: Raman Azad and John Neal; Sir Charles Gairdner Hospital, Nedlands: Paul W. Woods and Brigit L. Roberts; Concord Hospital, Concord: Mark R. Kol and Helen S. Wong. Austria: General Hospital of Vienna/Medical University of Vienna, Vienna: Katharina C. Riss and Thomas Staudinger. Belgium: Cliniques Universitaires St Luc, Université Catholique de Louvain, Brussels: Xavier Wittebole and Caroline Berghe; Center Hospitalier Universitaire DinantGodinne, Yvoir: Pierre A. Bulpa and Alain M. Dive; Acuut Ziekenhuis Sint Augustinus Veurne, Veurne: Rik Verstraete and Herve Lebbinck; Ghent University Hospital, Ghent: Pieter Depuydt and Joris Vermassen; University Hospitals Leuven, Leuven: Philippe Meersseman and Helga Ceunen. Brazil: Hospital Renascentista, Pouso Alegre: Jonas I. Rosa and Daniel O. Beraldo; Vitoria Apart Hospital, Serra: Claudio Piras and Adenilton M. Rampinelli; Hospital Das Clinicas, São Paulo: Antonio P. Nassar, Jr; Hospital Geral Do Grajaù, São Paulo: Sergio Mataloun and Marcelo Moock; Evangelical Hospital, Cachoeiro de Itapemirim/Espírito Santo: Marlus M. Thompson and Claudio H. Gonçalves; Hospital Moinhos de Vento, Porto Alegre: Ana Carolina P. Antônio and Aline Ascoli; Hospital Alvorada Taguatinga, Taguatinga: Rodrigo S. Biondi and Danielle C. Fontenele; Complexo Hospitalar Mngabeira Tarcisio Burity, Joao Pessoa: Danielle Nobrega and Vanessa M. Sales. Brunei: Raja Isteri Pengiran Anak Saleha Hospital, Bandar Seri Begawan: Ahmad Yazid Bin HJ Abul Wahab, Maizatul Ismail and Suresh Shindhe. Canada: Medical-Surgical Intensive Care Unit of St Michael's Hospital, Toronto: John Laffey and Francois Beloncle; St Joseph's Health Center, Toronto: Kyle G. Davies and Rob Cirone; Sunnybrook Health Sciences Center, Toronto: Venika Manoharan and Mehvish Ismail; Toronto Western Hospital, Toronto: Ewan C. Goligher and Mandeep Jassal; Medical Surgical Intensive Care Unit of the Toronto General Hospital, Toronto: Erin Nishikawa and Areej Javeed; Cardiovascular Intensive Care Unit of St Michael's Hospital, Toronto: Gerard Curley and Nuttapol Rittayamai; Cardiovascular Intensive Care Unit of the Toronto General Hospital, Toronto: Matteo Parotto and Niall D.

Ferguson; Mount Sinai Hospital, Toronto: Sangeeta Mehta and Jenny Knoll; Trauma-Neuro Intensive Care Unit of St Michael's Hospital, Toronto: Antoine Pronovost and Sergio Canestrini. Chile: Hospital Clínico Pontificia Universidad Católica de Chile, Santiago: Alejandro R. Bruhn and Patricio H. Garcia; Hospital Militar de Santiago, Santiago: Felipe A. Aliaga and Pamela A. Farías; Clinica Davila, Santiago: Jacob S. Yumha; Hospital Guillermo Grant Benavente, Concepcion: Claudia A. Ortiz and Javier E. Salas; Clinica Las Lilas, Santiago: Alejandro A. Saez and Luis D. Vega; Hospital Naval Almirante Nef, Viña del Mar: Eduardo F. Labarca and Felipe T. Martinez; Hospital Luis Tisné Brousse, Penanolen: Nicolás G. Carreño and Pilar Lora. China: Second Affiliated Hospital of Harbin Medical University, Harbin: Haitao Liu; Nanjing Zhong-da Hospital, Southeast University, Nanjing: Haibo Qiu and Ling Liu; First Affiliated Hospital of Anhui Medical University, Hefei: Rui Tang and Xiaoming Luo; Peking University People's Hospital, Beijing: Youzhong An and Huiying Zhao; Fourth Affiliated Hospital of Harbin Medical University, Harbin: Yan Gao and Zhe Zhai; Nanjing Jiangbei People's Hospital Affiliated to Medical School of Southeast University, Nanjing: Zheng L. Ye and Wei Wang; First Affiliated Hospital of Dalian Medical University, Dalian: Wenwen Li and Qingdong Li; Subei People's Hospital of Jiangsu Province, Yanghzou: Ruiqiang Zheng; Jinling Hospital, Nanjing: Wenkui Yu and Juanhong Shen; Urumqi General Hospital, Urumqi: Xinyu Li; Intensive Care Unit, First Affiliated Hospital of Wanna Medical College, Yijishan Hospital, Wuhu: Tao Yu and Weihua Lu; Sichuan Provincial People's Hospital, Chengdu: Ya Q. Wu and Xiao B. Huang; Hainan Province People's Hospital, Haikou: Zhenyang He; People's Hospital of Jiangxi Province, Nanchang: Yuanhua Lu; Qilu Hospital of Shandong University, Jinan: Hui Han and Fan Zhang; Zhejiang Provincial People's Hospital, Hangzhou: Renhua Sun; First Affiliated Hospital of Bengbu Medical College, Bengbu, Anhui: Hua X. Wang and Shu H. Qin; Nanjing Municipal Government Hospital, Nanjing: Bao H. Zhu and Jun Zhao; First Hospital of Lanzhou University, Lanzhou: Jian Liu and Bin Li; First Affiliated Hospital of Chongqing University of Medical Science, Chongqing: Jing L. Liu and Fa C. Zhou; Xuzhou Central Hospital, Xuzhou: Qiong J. Li and Xing Y. Zhang; First People's Hospital of Foshan, Foshan: Zhou Li-Xin and Qiang Xin-Hua; First Affiliated Hospital of Guangxi Medical University, Nanning: Liangyan Jiang; Renji Hospital, Shanghai Jiao Tong University School of Medicine, Shanghai: Yuan N. Gao and Xian Y. Zhao; First Hospital of Shanxi Medical University, Taiyuan: Yuan Y. Li and Xiao L. Li; Shandong Provincial Hospital, Jinan: Chunting Wang and Qingchun Yao; Fujian Provincial Hospital, Fuzhou: Rongguo Yu and Kai Chen; Henan Provincial People's Hospital, Zhengzhou: Huanzhang Shao and Bingyu Qin; Second Affiliated Hospital of Kunming Medical University, Kunming City: Qing

Q. Huang and Wei H. Zhu; Xiangya Hospital, Central South University, Changsha: Ai Y. Hang and Ma X. Hua; First Affiliated Hospital of Guangzhou Medical University, Guangzhou: Yimin Li and Yonghao Xu; People's Hospital of Hebei Province, Shijiazhuang: Yu D. Di and Long L. Ling; Guangdong General Hospital, Guangzhou: Tie H. Qin and Shou H. Wang; Beijing Tongren Hospital, Beijing: Junping Qin; Jiangsu Province Hospital, Nanjing: Yi Han and Suming Zhou. Colombia: Fundación Valle del Lili, Cali: Monica P. Vargas. Costa Rica: Hospital San Juan De Dios, San Jose: Juan I. Silesky Jimenez, Manuel A. González Rojas, Jaime E. SolisQuesada and Christian M. Ramirez-Alfaro. Czech Republic: University Hospital of Ostrava, Ostrava: Jan Máca and Peter Sklienka. Denmark: Aarhus Universitetshospital, Aarhus: Jakob Gjedsted and Aage Christiansen; Rigshopitalet: Jonas Nielsen. Ecuador: Hospital Militar, Quito: Boris G. Villamagua and Iguel Llano. France: Clinique du Millenaire, Montpellier: Philippe Burtin and Gautier Buzancais; Center Hospitalier, Roanne: Pascal Beuret and Nicolas Pelletier; Center Hospitalier Universitaire d'Angers, Angers: Satar Mortaza and Alain Mercat; Hôpital Marc Jacquet, Melun: Jonathan Chelly and Sébastien Jochmans; Center Hospitalier Universitaire Caen, Caen: Nicolas Terzi and Cédric Daubin; Henri Mondor Hospital, Créteil: Guillaume Carteaux and Nicolas de Prost; Cochin Hospital, Paris: Jean-Daniel Chiche and Fabrice Daviaud; Hôpital Tenon, Paris: Tài Pham and Muriel Fartoukh; CH Mulhouse-Emile Muller, Mulhouse: Guillaume Barberet and Jerome Biehler; Archet 1 University Hospital, Nice: Jean Dellamonica and Denis Doyen; Hopital Sainte Musse, Toulon: Jean-Michel Arnal and Anais Briquet; Hopital Nord–Réanimation des Détresses Respiratoires et Infections Sévères, Marseille: Sami Hraiech and Laurent Papazian; Hôpital Européen Georges Pompidou, Paris: Arnaud Follin; Louis Mourier Hospital, Colombes: Damien Roux and Jonathan Messika; Center Hospitalier de Dax, Dax: Evangelos Kalaitzis; Réanimation Médicale, Groupe Hospitalier Pitié-Salpêtrière, Paris: Laurence Dangers and Alain Combes; Assistance Publique-Hôpitaux de Paris Ambroise Paré, Boulogne-Billancourt: Siu-Ming Au; University Hospital Rouen, Rouen: Gaetan Béduneau and Dorothée Carpentier; Center Hospitalier Universitaire Amiens, Amiens–Salouel: Elie H. Zogheib and Herve Dupont; Center Hospitalier Intercommunal Robert Ballanger, Aulnay-sousBois: Sylvie Ricome and Francesco L. Santoli; Center Hospitalier René Dubos, Pontoise: Sebastien L. Besset; Center Hospitalier Intercommunal Portes de l'Oise, Beaumont-sur-Oise: Philippe Michel and Bruno Gelée; Archet 2 University Hospital, Nice: Pierre-Eric Danin and Bernard Goubaux; Center Hospitalier Pierre Oudot, Bourgoin Jallieu: Philippe J. Crova and Nga T. Phan; Center Hospitalier Dunkerque, Dunkerque: Frantz Berkelmans; Center Hospitalier de Belfort Montbéliard, Belfort: Julio C. Badie and Romain Tapponnier; Center Hospitalier Emile Muller, Mulhouse:

Josette Gally and Samy Khebbeb; Hôpital de Hautepierre-Hôpitaux Universitaires de Strasbourg, Strasbourg: Jean-Etienne Herbrecht and Francis Schneider; Center Hospitalier de Dieppe, Dieppe: PierreLouis M. Declercq and Jean-Philippe Rigaud; Bicetre, Le Kremin–Bicetre: Jacques Duranteau and Anatole Harrois; Center Hospitalier Universitaire Gabriel Montpied, Clermont-Ferrand: Russell Chabanne and Julien Marin; Center Hospitalier Universitaire Estaing, Clermont-Ferrand: Charlene Bigot and Sandrine Thibault; Center Hospitalier Intercommunal Eure-Seine Evreux, Evreux: Mohammed Ghazi and Messabi Boukhazna; Center Hospitalier de Châlons en Champagne, Châlons en Champagne: Salem Ould Zein; CH Beauvais, Beauvais: Jack R. Richecoeur and Daniele M. Combaux; Center Hospitalier Le Mans, Le Mans: Fabien Grelon and Charlene Le Moal; Hôpital Fleyriat, Bourg-en-Bresse: Elise P. Sauvadet and Adrien Robine; Hôpital Saint Louis, Paris: Virginie Lemiale and Danielle Reuter; Service de Pneumologie Pitié-Salpétrière, Paris: Martin Dres and Alexandre Demoule; Center Hospitalier Gonesse, Gonesse: Dany Goldgran-Toledano; Hôpital Croix Rousse, Lyon: Loredana Baboi and Claude Guérin. Germany: St Nikolaus-Stiftshospital, Andernach: Ralph Lohner; Fachkrankenhaus Coswig Gmbh, Coswig: Jens Kraßler and Susanne Schäfer; University Hospital Frankfurt, Frankfurt am Main: Kai D. Zacharowski and Patrick Meybohm; Department of Anesthesia and Intensive Care Medicine, University Hospital of Leipzig, Leipzig: Andreas W. Reske and Philipp Simon; Asklepios Klinik Langen, Langen: HansBernd F. Hopf and Michael Schuetz; Städtisches Krankenhaus Heinsberg, Heinsberg: Thomas Baltus. Greece: Hippokrateion General Hospital of Athens, Athens: Metaxia N. Papanikolaou and Theonymfi G. Papavasilopoulou; Gh Ahepa, Thessaloniki: Giannis A. Zacharas and Vasilis Ourailogloy; Hippokration General Hospital of Thessaloniki, Thessaloniki: Eleni K. Mouloudi and Eleni V. Massa; Hospital General of Kavala, Kavala: Eva O. Nagy and Electra E. Stamou; Papageorgiou General Hospital, Thessaloniki: Ellada V. Kiourtzieva and Marina A. Oikonomou. Guatemala: Hospital General de Enfermedades, Instituto Guatemalteco de Seguridad Social, Ciudad de Guatemala: Luis E. Avila; Centro Médico Militar, Guatemala: Cesar A. Cortez and Johanna E. Citalán. India: Deenanath Mangeshkar Hospital and Research Center, Pune: Sameer A. Jog and Safal D. Sable; Care Institute of Medical Sciences Hospital, Ahmedabad: Bhagyesh Shah; Sanjay Gandhi Postgraduate Institute of Medical Sciences, Lucknow: Mohan Gurjar and Arvind K. Baronia; Rajasthan Hospital, Ahmedabad: Mohammedfaruk Memon; National Institute of Mental Health and Neuro Sciences, Bangalore: Radhakrishnan Muthuchellappan and Venkatapura J. Ramesh; Anesthesiology Unit of the Kasturba Medical College and Department of Respiratory Therapy, School of Allied Health Sciences, Manipal University, Manipal: Anitha Shenoy and Ramesh Unnikrishnan; Sanjeevan Hospital, Pune: Subhal

B. Dixit and Rachana V. Rhayakar; Apollo Hospitals, Chennai: Nagarajan Ramakrishnan and Vallish K. Bhardwaj; Medicine Unit of the Kasturba Medical College and Department of Respiratory Therapy, School of Allied Health Sciences, Manipal University, Manipal: Heera L. Mahto and Sudha V. Sagar; G. Kuppuswamy Naidu Memorial Hospital, Coimbatore: Vijayanand Palaniswamy and Deeban Ganesan. Iran: National Research Institute of Tuberculosis and Lung Disease/Masih Daneshvari, Tehran: Seyed Mohammadreza Hashemian and Hamidreza Jamaati; Milad Hospital, Tehran: Farshad Heidari. Ireland: St Vincent's University Hospital, Dublin: Edel A. Meaney and Alistair Nichol; Mercy University Hospital, Cork: Karl M. Knapman and Donall O'Croinin; Cork University Hospital, Cork: Eimhin S. Dunne and Dorothy M. Breen; Galway University Hospital, Galway: Kevin P. Clarkson and Rola F. Jaafar; Beaumont Hospital, Dublin: Rory Dwyer and Fahd Amir; Mater Misericordiae University Hospital, Dublin: Olaitan O. Ajetunmobi and Aogan C. O'Muircheartaigh; Tallaght Hospital, Dublin: Colin S. Black and Nuala Treanor; Saint James's Hospital, Dublin: Daniel V. Collins and Wahid Altaf. Italy: Santa Maria delle Croci Hospital, Ravenna: Gianluca Zani and Maurizio Fusari; Arcispedale Sant'Anna Ferrara, Ferrara: Savino Spadaro and Carlo A. Volta; Ospedale Profili, Fabriano, Ancona: Romano Graziani and Barbara Brunettini; Umberto I. Nocera Inferiore, Nocera Inferiore Salerno: Salvatore Palmese; Azienda Ospedaliera San Paolo–Polo Universitario–Università degli Studi di Milano, Milan: Paolo Formenti and Michele Umbrello; Sant'Anna, San Fermo Della Battaglia, Como: Andrea Lombardo; Spedali Civili Brescia, Brescia: Elisabetta Pecci and Marco Botteri; Fondazione Istituto di Ricovero e Cura a Carattere Scientifico Ca Granda, Ospedale Maggiore Policlinico, Milan: Monica Savioli and Alessandro Protti; University Campus Bio-Medico of Rome, Rome: Alessia Mattei and Lorenzo Schiavoni; Azienda Ospedaliera "Mellino Mellini", Chiari, Brescia: Andrea Tinnirello and Manuel Todeschini; Policlinico P. Giaccone, University of Palermo, Palermo: Antonino Giarratano and Andrea Cortegiani; Niguarda Cà Granda Hospital, Milan: Sara Sher and Anna Rossi; A. Gemelli University Hospital, Rome: Massimo M. Antonelli and Luca M. Montini; Ospedale "Sandro Pertini", Rome: Paolo Casalena and Sergio Scafetti; Istituto Mediterraneo per i Trapianti e Terapie ad Alta Specializzazione; Istituto Di Ricovero e Cura a Carattere Scientifico; University of Pittsburgh Medical Center, Palermo: Giovanna Panarello and Giovanna Occhipinti; Ospedale San Gerardo, Monza: Nicolò Patroniti and Matteo Pozzi; Santa Maria Della Scaletta, Imola: Roberto R. Biscione and Michela M. Poli; Humanitas Research Hospital, Rozzano: Ferdinando Raimondi and Daniela Albiero; Ospedale Desio-Ao Desio-Vimercate, Desio: Giulia Crapelli and Eduardo Beck; Pinetagrande Private Hospital, Castelvolturno: Vincenzo Pota and Vincenzo Schiavone; Istituto di Ricovero e Cura a

Carattere Scientifico San Martino Ist, Genova: Alexandre Molin and Fabio Tarantino; Ospedale San Raffaele, Milano: Giacomo Monti and Elena Frati; Ospedali Riuniti Di Foggia, Foggia: Lucia Mirabella and Gilda Cinnella; Azienda Ospedaliera Luigi Sacco–Polo Universitario, Milano: Tommaso Fossali and Riccardo Colombo; Azienda Ospedaliero Universitaria Città della Salute e della Scienza di Torino, Turin: Pierpaolo Terragni and Ilaria Pattarino; Università degli Studi di Pavia-Fondazione Istituto di Ricovero e Cura a Carattere Scientifico Policlinico San Matteo, Pavia: Francesco Mojoli and Antonio Braschi; Ao Ospedale Civile Legnano, Legnano: Erika E. Borotto; Arnas Ospedale Civico Di Cristina Benfratelli, Palermo: Andrea N. Cracchiolo and Daniela M. Palma; Azienda Ospedaliera Della Provincia Di Lecco–Ospedale "A. Manzoni", Lecco: Francesco Raponi and Giuseppe Foti; A.O. Provincia Di Lecco–Ospedale Alessandro Manzoni, Lecco: Ettore R. Vascotto and Andrea Coppadoro; Cliniche Universitarie Sassari, Sassari: Luca Brazzi and Leda Floris; Istituto di Ricovero e Cura a Carattere Scientifico Policlinico San Matteo, Pavia: Giorgio A. Iotti and Aaron Venti. Japan: Yokohama City University Hospital, Yokohama: Osamu Yamaguchi and Shunsuke Takagi; Toyooka Hospital, Toyooka City: Hiroki N. Maeyama; Chiba University Hospital, Chiba City: Eizo Watanabe and Yoshihiro Yamaji; Okayma University Hospital, Okayama: Kazuyoshi Shimizu and Kyoko Shiozaki; Japanese Foundation for Cancer Research, Cancer Institute Hospital, Department of Emergency Medicine and Critical Care, Tokyo: Satoru Futami; Ibaraki Prefectural Central Hospital, Kasama: Sekine Ryosuke; Tohoku University Hospital, Sendai-Shi: Koji Saito and Yoshinobu Kameyama; Tokyo Medical University Hachioji Medical Center, Hachioji, Tokyo: Keiko Ueno; Tokushima University Hospital, Tokushima: Masayo Izawa and Nao Okuda; Maebashi Red Cross Hospital, Gunma Maebashi: Hiroyuki Suzuki and Tomofumi Harasawa; Urasoe General Hospital, Urasoe: Michitaka Nasu and Tadaaki Takada; Ohta General Hospital Foundation Ohta Nishinouchi Hospital, Fukushima: Fumihito Ito; Jichi Medical University Hospital, Shimotsuke: Shin Nunomiya and Kansuke Koyama; Mito Kyodo General Hospital, Tsukuba University Hospital Mito Medical Center, Mito: Toshikazu Abe; Sendai City Hospital, Sendai: Kohkichi Andoh and Kohei Kusumoto; Ja Hiroshima General Hospital, Hatsukaichi City, Hiroshima: Akira Hirata and Akihiro Takaba; Yokohama Rosai Hospital, Yokohama: Hiroyasu Kimura; Nagasaki University Hospital, Nagasaki: Shuhei Matsumoto and Ushio Higashijima; Niigata University Medical and Dental Hospital, Niigata: Hiroyuki Honda and Nobumasa Aoki; Mie University Hospital, Tsu, Mie: Hiroshi Imai; Yamaguchi University Hospital, Ube, Yamaguchi: Yasuaki Ogino and Ichiko Mizuguchi; Saiseikai Kumamoto Hospital, Kumamoto City: Kazuya Ichikado; Shinshu University School of Medicine, Matsumoto City: Kenichi Nitta and Katsunori

Mochizuki; Kuki General Hospital, Kuki: Tomoaki Hashida; Kyoto Medical Center, Kyoto: Hiroyuki Tanaka; Fujita Health University, Toyoake: Tomoyuki Nakamura and Daisuke Niimi; Rakwakai Marutamachi Hospital, Kyoto: Takeshi Ueda; Osaka University Hospital, Suita City, Osaka Prefecture: Yozo Kashiwa and Akinori Uchiyama. Latvia: Paul Stradins Clinical University Hospital, Riga: Olegs Sabelnikovs and Peteris Oss. Lebanon: Kortbawi Hospital, Jounieh: Youssef Haddad. Malaysia: Hospital Kapit, Kapit: Kong Y. Liew. Mexico: Instituto Nacional de Cancerología, Mexico City: Silvio A. Ñamendys-Silva and Yves D. Jarquin-Badiola; Hospital de Especialidades "Antonio Fraga Mouret" Centro Medico Nacional La Raza Instituto Mexicano del Seguro Social, Mexico City: Luis A. Sanchez-Hurtado and Saira S. Gomez-Flores; Hospital Regional 1° de Octubre, Mexico City: Maria C. Marin and Asisclo J. Villagomez; Hospital General Dr. Manuel Gea Gonzalez, Mexico City: Jordana S. Lemus and Jonathan M. Fierro; Hospital General de Zona No. 1 Instituto Mexicano del Seguro Social Tepic Nayarit, Tepic: Mavy Ramirez Cervantes and Francisco Javier Flores Mejia; Centro Medico Dalinde, Mexico City: Dulce Dector and Alejandro Rojas; Opd Hospital Civil de Guadalajara Hospital Juan I. Menchaca, Guadalajara: Daniel R. Gonzalez and Claudia R. Estrella; Hospital Regional de Ciudad Madero Pemex, Ciudad Madero: Jorge R. Sanchez-Medina and Alvaro Ramirez-Gutierrez; Centro Médico American British Cowdray, Mexico City: Fernando G. George and Janet S. Aguirre; Hospital Juarez de Mexico, Mexico City: Juan A. Buensuseso and Manuel Poblano. Morocco: Mohammed V University, University Teaching Ibn Sina Hospital, Rabat: Tarek Dendane and Amine Ali Zeggwagh; Hopital Militaire D'Instruction Mohammed V, Rabat: Hicham Balkhi; Errazi, Marrakech: Mina Elkhayari and Nacer Samkaoui; University Teaching Hospital Ibn Rushd, Casablanca: Hanane Ezzouine and Abdellatif Benslama; Hôpital des Spécialités de Rabat, Rabat: Mourad Amor and Wajdi Maazouzi. The Netherlands: Tjongerschans, Heerenveen: Nedim Cimic and Oliver Beck; Cwz, Nijmegen: Monique M. Bruns and Jeroen A. Schouten; Rijnstate Hospital, Arnhem: Myra Rinia and Monique Raaijmakers; Radboud Umc, Nijmegen: Leo M. Heunks and Hellen M. Van Wezel; Maastricht University Medical Center, Maastricht: Serge J. Heines and Ulrich Strauch; Catharinaziekenhuis, Eindhoven: Marc P. Buise; Academic Medical Center, Amsterdam: Fabienne D. Simonis and Marcus J. Schultz. New Zealand: Tauranga Hospital, Tauranga: Jennifer C. Goodson and Troy S. Browne; Wellington Hospital, Wellington: Leanlove Navarra and Anna Hunt; Dunedin Hospital, Dunedin: Robyn A. Hutchison and Mathew B. Bailey; Auckland City Hospital, Auckland: Lynette Newby and Colin McArthur; Whangarei Base Hospital, Whangarei: Michael Kalkoff and Alex Mcleod; North Shore Hospital, Auckland: Jonathan Casement and Danielle J. Hacking. Norway: Ålesund Hospital,

Ålesund: Finn H. Andersen and Merete S. Dolva; Oslo University Hospital, Rikshospitalet Medical Center, Oslo: Jon H. Laake and Andreas Barratt-Due; Stavanger University Hospital, Stavanger: Kim Andre L. Noremark and Eldar Søreide; Haukeland University Hospital, Bergen: Brit Å. Sjøbø and Anne B. Guttormsen. Peru: Hospital Nacional Edgardo Rebagliati Martins, Lima: Hector H. Leon Yoshido; Clínica Ricardo Palma, Lima: Ronald Zumaran Aguilar and Fredy A. Montes Oscanoa. Philippines: The Medical City, Pasig: Alain U. Alisasis and Joanne B. Robles; Chong Hua Hospital, Cebu: Rossini Abbie B. Pasanting-Lim and Beatriz C. Tan. Poland: Warsaw University Hospital, Warsaw: Pawel Andruszkiewicz and Karina Jakubowska. Portugal: Centro Hospitalar Da Cova Da Beira, Covilhã: Cristina M. Coxo; Hospital Santa Maria, Chln, Lisboa: António M. Alvarez and Bruno S. Oliveira; Centro Hospitalar Trás-Os-Montes E. Alto Douro, Hospital de S. Pedro-Vila Real, Vila Real: Gustavo M. Montanha and Nelson C. Barros; Hospital Beatriz Ângelo, Loures: Carlos S. Pereira and António M. Messias; Hospital de Santa Maria, Lisboa: Jorge M. Monteiro; Centro Hospitalar Médio Tejo–Hospital de Abrantes, Abrantes: Ana M. Araujo and Nuno T. Catorze; Instituto Português de Oncologia de Lisboa, Lisboa: Susan M. Marum and Maria J. Bouw; Hospital Garcia de Orta, Almada: Rui M. Gomes and Vania A. Brito; Centro Hospitalar Do Algarve, Faro: Silvia Castro and Joana M. Estilita; Hospital de Cascais, Alcabideche: Filipa M. Barros; Hospital Prof. Doutor Fernando Fonseca Epe, Amadora: Isabel M. Serra and Aurelia M. Martinho. Romania: Fundeni Clinical Institute, Bucharest: Dana R. Tomescu and Alexandra Marcu; Emergency Clinical County Hospital Timisoara, Timisoara: Ovidiu H. Bedreag and Marius Papurica; Elias University Emergency Hospital, Bucharest: Dan E. Corneci and Silvius Ioan Negoita. Russian Federation: University Hospital, Kemerovo: Evgeny Grigoriev; Krasnoyarsk Regional Hospital, Krasnoyarsk State Medical University, Krasnoyarsk: Alexey I. Gritsan and Andrey A. Gazenkampf. Saudi Arabia: General Intensive Care Unit of Prince Sultan Military Medical City, Riyadh: Ghaleb Almekhlafi and Mohamad M. Albarrak; Surgical Intensive Care Unit of Prince Sultan Military Medical City, Riyadh: Ghanem M. Mustafa; King Faisal Hospital and Research Center, Riyadh: Khalid A. Maghrabi and Nawal Salahuddin; King Fahad Hospital, Baha: Tharwat M. Aisa; Neuro Critical Care Unit, King Abdulaziz Medical City, Riyadh: Ahmed S. Al Jabbary and Edgardo Tabhan; Intensive Care Unit, King Abdulaziz Medical City, Riyadh: Yaseen M. Arabi; Surgical Intensive Care Unit, King Abdulaziz Medical City, Riyadh: Yaseen M. Arabi and Olivia A. Trinidad; Trauma Intensive Care Unit, King Abdulaziz Medical City, Riyadh: Hasan M. Al Dorzi and Edgardo E. Tabhan. Serbia: Clinical Center of Serbia, Belgrade: Vesna Bumbasirevic and Bojan Jovanovic. South Africa: Charlotte Maxeke Johannesburg Academic Hospital, Johannesburg: Stefan Bolon and Oliver

Smith. Spain: Hospital Sant Pau, Barcelona: Jordi Mancebo and Hernan Aguirre-Bermeo; Hospital Universitari Bellvitge, L'Hospitalet de Llobregat, Barcelona: Juan C. Lopez-Delgado and Francisco Esteve; Hospital Son Llatzer, Palma de Mallorca: Gemma Rialp and Catalina Forteza; Sabadell Hospital, Centro de Investigación Biomédica en Red Enfermedades Respiratorias, Sabadell: Candelaria De Haro and Antonio Artigas; Hospital Universitario Central de Asturias, Oviedo: Guillermo M. Albaiceta and Sara De Cima-Iglesias; Complejo Hospitalario Universitario A Coruña, A Coruña: Leticia Seoane-Quiroga and Alexandra Ceniceros-Barros; Hospital Universitario Miguel Servet, Zaragoza: Antonio L. RuizAguilar and Luis M. Claraco-Vega; Morales Meseguer University Hospital, Murcia: Juan Alfonso Soler and Maria del Carmen Lorente; Hospital Universitario del Henares, Coslada: Cecilia Hermosa and Federico Gordo; Complejo Asistencial de Palencia, Hospital Rio Carrión, Palencia: Miryam PrietoGonzález and Juan B. López-Messa; Fundación Jiménez Díaz, Madrid: Manuel P. Perez and Cesar P. Perez; Hospital Clínico Universitario Lozano Blesa, Zaragoza: Raquel Montoiro Allue; Hospital Verge de la Cinta, Tortosa: Ferran RocheCampo and Marcos Ibañez-Santacruz; Hospital Universitario 12 de Octubre, Madrid: Susana Temprano; Hospital Universitario Príncipe de Asturias, Alcalá de Henares, Madrid: Maria C. Pintado and Raul De Pablo; Hospital Universitari Germans Trias I Pujol, Badalona: Pilar Ricart Aroa Gómez; Hospital Universitario Arnau de Vilanova de Lleida, Lleida: Silvia Rodriguez Ruiz and Silvia Iglesias Moles; Cst Terrassa, Barcelona: Mª Teresa Jurado and Alfons Arizmendi; Hospital Universitari Mútua Terrassa, Terrassa: Enrique A. Piacentini; Hospital Universitario de Móstoles, Mostoles: Nieves Franco and Teresa Honrubia; Complejo Asistencial de Salamanca, Salamanca: Meisy Perez Cheng and Elena Perez Losada; Hospital General Universitario de Ciudad Real, Ciudad Real: Javier Blanco and Luis J. Yuste; Torrecardenas, Almeria: Cecilia Carbayo-Gorriz and Francisca G. Cazorla-Barranquero; Hospital Universitario Donostia, San Sebastian: Javier G. Alonso and Rosa S. Alda; Hospital Universitario de Torrejón, Madrid: Ángela Algaba and Gonzalo Navarro; Hospital Universitario de La Princesa, Madrid: Enrique Cereijo and Esther Diaz-Rodriguez; Hospital Universitario Lucus Augusti, Lugo: Diego Pastor Marcos and Laura Alvarez Montero; Hospital Universitario Santa Lucia, Cartagena: Luis Herrera Para and Roberto Jimenez Sanchez; Hospital Universitario Severo Ochoa, Leganes, Madrid: Miguel Angel Blasco Navalpotro and Ricardo Diaz Abad; University Hospital of Nuestra Señora de Candelaria, Santa Cruz de Tenerife: Raquel Montiel González and Dácil Parrilla Toribio; Hospital Universitario Marques de Valdecilla, Santander: Alejandro G. Castro and Maria Jose D. Artiga; Hospital Infanta Cristina, Parla, Madrid: Oscar Penuelas; Hospital General de Catalunya, Sant Cugat del Valles: Tomas P. Roser and

Moreno F. Olga; San Pedro de Alcántara, Cáceres: Elena Gallego Curto and Rocío Manzano Sánchez; Sant Joan de Reus, Reus: Vallverdu P. Imma and Garcia M. Elisabet; Hospital Joan XXIII, Tarragona: Laura Claverias and Monica Magret; Hospital Universitario de Getafe, Madrid: Ana M. Pellicer and Lucia L. Rodriguez; Hospital Universitario Río Hortega, Valladolid: Jesús Sánchez-Ballesteros and Ángela González-Salamanca; Hospital Arquitecto Marcide, Ferrol, La Coruña: Antonio G. Jimenez and Francisco P. Huerta; Hospital General Universitario Gregorio Marañón, Madrid: Juan Carlos J. Sotillo Diaz and Esther Bermejo Lopez; Hospital General de Segovia, Segovia: David D. Llinares Moya and Alec A. Tallet Alfonso; Hospital General Universitario Reina Sofia, Murcia: Palazon Sanchez Eugenio Luis and Palazon Sanchez Cesar; Complejo Hospitalario Universitario de Albacete, Albacete: Sánchez I. Rafael and Corcoles G. Virgilio; Hospital Infanta Elena, Valdemoro: Noelia N. Recio. Sweden: Sahlgrenska University Hospital, Gothenburg: Richard O. Adamsson and Christian C. Rylander; Karolinska University Hospital, Stockholm: Bernhard Holzgraefe and Lars M. Broman; Akademiska Sjukhuset Uppsala, Uppsala: Joanna Wessbergh and Linnea Persson; Vrinnevisjukhuset, Norrköping: Fredrik Schiöler and Hans Kedelv; Linkoping University Hospital, Linköping: Anna Oscarsson Tibblin and Henrik Appelberg; Skellefteå Lasarett, Skellefteå: Lars Hedlund and Johan Helleberg; Karolinska University Hospital Solna, Stockholm: Karin E. Eriksson and Rita Glietsch; Umeå University Hospital, Umeå: Niklas Larsson and Ingela Nygren; Danderyd Hospital, Stockholm: Silvia L. Nunes and Anna-Karin Morin; Lund University Hospital, Lund: Thomas Kander and Anne Adolfsson. Switzerland: Centre Hospitalier Universitaire Vaudois, Lausanne: Lise Piquilloud; Hôpital Neuchâtelois–La Chaux de-Fonds, La Chaux-de-Fonds: Hervé O. Zender and Corinne Leemann-Refondini. Tunisia: Hopital Taher Sfar Mahdia, Mahdia: Souheil Elatrous; University Hospital Farhat Hached Sousse, Sousse: Slaheddine Bouchoucha and Imed Chouchene; Center Hospitalier Universitaire F. Bourguiba, Monastir: Islem Ouanes; Mongi Slim University Hospital, La Marsa: Asma Ben Souissi and Salma Kamoun. Turkey: Cerrahpasa Medical Faculty Emergency Intensive Care Unit, Istanbul: Oktay Demirkiran; Cerrahpasa Medical Faculty Sadi Sun Intensive Care Unit, Istanbul: Mustafa Aker and Emre Erbabacan; Uludag University Medical Faculty, Bursa: Ilkay Ceylan and Nermin Kelebek Girgin; Ankara University Faculty of Medicine, Reanimation 3rd Level Intensive Care Unit, Ankara: Menekse Ozcelik and Necmettin Ünal; Ankara University Faculty of Medicine, 2nd Level Intensive Care Unit–Postoperative Intensive Care Unit, Ankara: Basak Ceyda Meco; Istanbul Kartal Egitim Ve Arastirma Hastanesi, Istanbul: Onat O. Akyol and Suleyman S. Derman. UK: Papworth Hospital, Cambridge: Barry Kennedy and Ken Parhar; Royal Glamorgan Hospital, Llantrisant: Latha Srinivasa; Royal Victoria

Hospital, Belfast: Lia McNamee and Danny McAuley; Jack Steinberg Intensive Care Unit of the King's College, London: Phil Hopkins and Clare Mellis; Frank Stansil Intensive Care Unit of the King's College Hospital, London: Vivek Kakar; Liver Intensive Care Unit of the King's College, London: Dan Hadfield; Christine Brown Intensive Care Unit of the King's College, London: Andre Vercueil; West Suffolk Hospital, Bury St Edmunds: Kaushik Bhowmick and Sally K. Humphreys; Craigavon Area Hospital, Portadown: Andrew Ferguson and Raymond Mckee; Barts Health National Health Service Trust, Whipps Cross Hospital, Leytonstone: Ashok S. Raj and Danielle A. Fawkes; Kettering General Hospital, Foundation National Health Service Trust, Northamptonshire: Philip Watt and Linda Twohey; Barnet General Hospital, Barnet: Rajeev R. Jha, Matthew Thomas, Alex Morton and Varsha Kadaba; Rotherham General Hospital, Rotherham: Mark J. Smith and Anil P. Hormis; City Hospital, Birmingham: Santhana G. Kannan and Miriam Namih; Poole Hospital National Health Service Foundation Trust, Poole: Henrik Reschreiter and Julie Camsooksai; Weston General Hospital, Weston-Super-Mare: Alek Kumar and Szabolcs Rugonfalvi; Antrim Area Hospital, Antrim: Christopher Nutt and Orla Oneill; Aintree University Hospital, Liverpool: Colette Seasman and Ged Dempsey; Northern General Hospital, Sheffield: Christopher J. Scott and Helen E. Ellis; John Radcliffe Hospital, Oxford: Stuart Mckechnie and Paula J. Hutton; St Georges Hospital, London: Nora N. Di Tomasso and Michela N. Vitale; Hillingdon Hospital, Uxbridge: Ruth O. Griffin and Michael N. Dean; Royal Bournemouth and Christchurch National Health Service Foundation Trust, Bournemouth: Julius H. Cranshaw and Emma L. Willett; Guy's and St Thomas' National Health Service Foundation Trust, London: Nicholas Ioannou; Guy's and St Thomas' Severe Respiratory Failure Service, Whittington Hospital, London: Sarah Gillis; Wexham Park Hospital, Slough: Peter Csabi; Western General Hospital, Edinburgh: Rosaleen Macfadyen and Heidi Dawson; Royal Preston Hospital, Preston: Pieter D. Preez and Alexandra J. Williams; Brighton and Sussex University Hospitals National Health Service Trust, Brighton: Owen Boyd and Laura Ortiz-Ruiz de Gordoa; East and North Herts National Health Service Trust, Stevenage: Jon Bramall and Sophie Symmonds; Barnsley Hospital, Barnsley: Simon K. Chau and Tim Wenham; Prince Charles Hospital, Merthyr Tydfil: Tamas Szakmany and Piroska Toth-Tarsoly; University Hospital of South Manchester National Health Service Foundation Trust, Manchester: Katie H. McCalman and Peter Alexander; Harrogate District Hospital, Harrogate: Lorraine Stephenson and Thomas Collyer; East and North Herts National Health Service Trust, Welwyn Garden City: Rhiannon Chapman and Raphael Cooper; Western Infirmary, Glasgow: Russell M. Allan and Malcolm Sim; Dumfries and Galloway Royal Infirmary, Dumfries: David W. Wrathall and Donald A.

Irvine; Charing Cross Hospital, London: Kim S. Zantua and John C. Adams; Worcestershire Royal Hospital, Worcester: Andrew J. Burtenshaw and Gareth P. Sellors; Royal Liverpool University Hospital, Liverpool: Ingeborg D. Welters and Karen E. Williams; Royal Alexandra Hospital, Glasgow: Robert J. Hessell and Matthew G. Oldroyd; Morriston Hospital, Swansea: Ceri E. Battle and Suresh Pillai; Frimley Park Hospital, Frimley: Istvan Kajtor and Mageswaran Sivashanmugavel; Altnagelvin Hospital, Derry: Sinead C. Okane and Adrian Donnelly; Buckinghamshire Healthcare National Health Service Trust, High Wycombe: Aniko D. Frigyik and Jon P. Careless; Milton Keynes Hospital, Milton Keynes: Martin M. May and Richard Stewart; Ulster Hospital, Belfast: T. John Trinder and Samantha J. Hagan; University Hospital of Wales, Cardiff: Jade M. Cole; Freeman Hospital, Newcastle upon Tyne: Caroline C. MacFie and Anna T. Dowling. Uruguay: Hospital Español, Montevideo: Javier Hurtado and Nicolás Nin; Cudam, Montevideo: Javier Hurtado; Sanatorio Mautone, Maldonado: Edgardo Nuñez; Sanatorio Americano, Montevideo: Gustavo Pittini and Ruben Rodriguez; Hospital de Clínicas, Montevideo: María C. Imperio and Cristina Santos; Circulo Católico Obreros Uruguay–Sanatorio Juan Pablo II, Montevido: Ana G. França and Alejandro Ebeid; Centro de Asistencia del Sindicato Médico del Uruguay, Montevideo: Alberto Deicas and Carolina Serra. USA: St. Louis University Hospital, St. Louis, Missouri: Aditya Uppalapati and Ghassan Kamel; Beth Israel Deaconess Medical Center, Boston, Massachusetts: Valerie M. Banner-Goodspeed and Jeremy R. Beitler; Memorial Medical Center, Springfield, Illinois: Satyanarayana Reddy Mukkera and Shreedhar Kulkarni; University of Cincinnati Medical Center, Cincinnati, Ohio: John O. Shinn III and Dina Gomaa; Massachusetts General Hospital, Boston, Massachusetts: Christopher Tainter, Jarone Lee and Tomaz MesarJarone Lee; R. Adams Cowley Shock Trauma Center, Baltimore, Maryland: Dale J. Yeatts and Jessica Warren; Intermountain Medical Center, Murray, Utah: Michael J. Lanspa, Russel R. Miller, Colin K. Grissom and Samuel M. Brown; Mayo Clinic, Rochester, Minnesota: Philippe R. Bauer; North Shore Medical Center, Salem, Massachusetts: Ryan J. Gosselin and Barrett T. Kitch; Albany Medical Center, Albany, New York: Jason E. Cohen, Scott H. Beegle and Shazia Choudry; John H. Stoger Hospital of Cook County, Chicago, Illinois: Renaud M. Gueret and Aiman Tulaimat; University of Alabama at Birmingham, Birmingham, Alabama: William Stigler and Hitesh Batra; Duke University Hospital, Durham, North Carolina: Nidhi G. Huff; Iowa Methodist Medical Center, Des Moines, Iowa: Keith D. Lamb and Trevor W. Oetting; Surgical and Neurosciences Intensive Care Unit of the University of Iowa Hospitals and Clinics, Iowa City, Iowa: Nicholas M. Mohr and Claine Judy; Medical Center of Louisiana at New Orleans, New Orleans, Louisiana: Shigeki Saito and Fayez M. Kheir; Tulane University, New

Orleans, Louisiana: Fayez Kheir; Critical Care Unit of the University of Iowa Hospitals and Clinics, Iowa City, Iowa: Adam B. Schlichting and Angela Delsing; University of California, San Diego Medical Center, San Diego, California: Daniel R. Crouch and Mary Elmasri; University of California San Diego Thornton Hospital, La Jolla, California: Daniel R. Crouch and Dina Ismail; University Hospital, Cincinnati, Ohio: Kyle R. Dreyer, Thomas C. Blakeman and Dina Gomaa; Tower 3B Medical Intensive Care Unit of Brigham and Women's Hospital, Boston, Massachusetts: Rebecca M. Baro and, Carolina Quintana Grijalba; Tower 8C Burn/Trauma Intensive Care Unit of Brigham and Women's Hospital, Boston, Massachusetts: Peter C. Hou; Tower 8D Surgical Intensive Care Unit of Brigham and Women's Hospital, Boston, Massachusetts: Raghu Seethala; Tower 9C Neurosurgical Intensive Care Unit of Brigham and Women's Hospital, Boston, Massachusetts: Imo Aisiku; Tower 9D Neurological Intensive Care Unit of Brigham and Women's Hospital, Boston, Massachusetts: Galen Henderson; Tower 11C Thoracic Intensive Care Unit of Brigham and Women's Hospital, Boston, Massachusetts: Gyorgy Frendl; Shapiro 6W Cardiac Surgery Intensive Care Unit of Brigham and Women's Hospital, Boston, Massachusetts: Sen-Kuang Hou; Shapiro 9E Coronary Care Unit of Brigham and Women's Hospital, Boston, Massachusetts: Robert L. Owens and Ashley Schomer.

Table S1. Differences between patients who received non-invasive and invasive ventilation as first-line respiratory support. Values represent median (interquartile range) or count (percentage). P-values were obtained using Wilcoxon or Chi-square tests (for quantitative and qualitative data respectively). PBW: Predicted Body Weight. COPD: Chronic Obstructive Pulmonary Disease. PEEP: Positive End-Expiratory Pressure. *SOFA score at day 1 was available for 292 patients.

	**Non-invasive ventilation (N=67)**	**Invasive ventilation (N=314)**	**p-value**
Gender			0.207
Female	32 (48%)	121 (39%)	
Male	35 (52%)	193 (61%)	
Age (year)	72 (56 - 81)	69 (59 - 77)	0.163
Predicted body weight (Kg)	58 (52 - 64)	60 (52 - 67)	0.414
Chronic heart failure	35 (52%)	127	0.102
Comorbidities			
Diabetes	28 (42%)	88 (28%)	0.038
Chronic kidney failure	13 (19%)	60 (19%)	1
Chronic liver failure	1 (1%)	7 (2%)	1
Solid neoplasm	0	11 (4%)	0.249
Hematological neoplasm	1 (1%)	5 (2%)	1
Chronic immunosuppression	1 (1%)	6 (2%)	1
COPD	9 (13%)	46 (15%)	0.948
Home ventilation	1 (1%)	5 (2%)	1
**Day 1**			
SOFA score	5 (4 – 7)	10 (8 – 12)	<0.001
Hemodynamic SOFA score*			<0.001
MAP≥70 mmHg	47 (73%)	64 (21%)	
MAP<70 mmHg	8 (13%)	32 (11%)	
Dopamine ≤ 5 µg/Kg/min or dobutamine	4 (6%)	21 (7%)	
Dopamine 5-15 µg/Kg/min or norepinephrine ≤ 0.1 µg/Kg/min or epinephrin ≤ 0.1 µg/Kg/min	2 (3%)	59 (20%)	
Dopamine > 15 µg/Kg/min or norepinephrine > 0.1 µg/Kg/min or epinephrin > 0.1 µg/Kg/min	3 (5%)	123 (41%)	
Arterial pH	7.38 (7.3 - 7.44)	7.35 (7.26 - 7.42)	0.164
PaO_2 / FiO_2	178 (117 – 236)	172 (115 – 230)	0.581
PaCO2(mmHg)	39 (33 – 46)	40 (35 – 48)	0.352
Tidal volume (ml/Kg PBW)	8.2 (7.4 – 11.1)	8.3 (7.2 – 9.4)	0.341
PEEP (cmH_2O)	7 (5 – 8)	6 (5 – 9)	0.940
Peak pressure (cmH_2O)	16 (13 – 20)	24 (20 – 30)	<0.001
Plateau pressure (cmH_2O)	16 (13 – 20)	21 (18 – 25)	<0.001

Driving pressure (cmH_2O)	9 (6 – 12)	14 (11 – 17)	<0.001
Respiratory rate (breaths/min)	22 (18 – 30)	18 (14 – 21)	<0.001
**ICU evolution**			
Length of mechanical ventilation (days)	5 (3 – 11)	4 (2 – 10)	0.712
ICU mortality	10 (15%)	124 (39%)	<0.001

Table S2. Differences between patients with not prolonged and prolonged invasive mechanical ventilation (more than 10 days). Values represent median (interquartile range) or count (percentage). P-values were obtained using Wilcoxon or Chi-square tests (for quantitative and qualitative data respectively). PBW: Predicted Body Weight. COPD: Chronic Obstructive Pulmonary Disease. PEEP: Positive End-Expiratory Pressure. *SOFA score at day 1 was available for 292 patients.

	**Not prolonged ventilation (N=261)**	**Prolonged ventilation (N=66)**	**p-value**
Gender			0.950
Female	104 (38%)	26 (44%)	
Male	157 (62%)	42 (56%)	
Age (year)	71 (60 - 78)	66 (60 - 76)	0.249
Predicted body weight (Kg)	60 (52 - 66)	61 (52 - 67)	0.414
Chronic heart failure	98 (38%)	31 (47%)	0.491
Comorbidities			
Diabetes	68 (26%)	21 (32%)	0.723
Chronic kidney failure	46 (18%)	11 (17%)	0.754
Chronic liver failure	5 (2%)	1 (1.5%)	1
Solid neoplasm	10 (4%)	2 (3%)	0.939
Hematological neoplasm	4 (1.5%)	1 (1.5%)	1
Chronic immunosuppression	3 (1%)	3 (5%)	0.232
COPD	34 (13%)	12 (18%)	0.560
Home ventilation	3 (1%)	2 (3%)	0.651
**Day 1**			
SOFA score	10 (8 – 12)	10 (9 – 12)	0.884
Hemodynamic SOFA score*			0.269
MAP≥70 mmHg	58 (26%)	9 (14%)	
MAP<70 mmHg	21 (9%)	9 (14%)	
Dopamine ≤ 5 µg/Kg/min or dobutamine	14 (6%)	5 (7%)	
Dopamine 5-15 µg/Kg/min or norepinephrine ≤ 0.1 µg/Kg/min or epinephrin ≤ 0.1 µg/Kg/min	44 (19%)	11 (17%)	
Dopamine > 15 µg/Kg/min or norepinephrine > 0.1 µg/Kg/min or epinephrin > 0.1 µg/Kg/min	90 (40%)	31 (48%)	
Arterial pH	7.35 (7.25 – 7.42)	7.38 (7.31 – 7.44)	0.068
PaO_2 / FiO_2	172 (118 - 232)	172 (115 - 240)	0.703
PaCO2(mmHg)	39 (34 - 47)	40 (34 - 47)	0.978
Tidal volume (ml/Kg PBW)	8.4 (7.4 - 9.5)	7.6 (6.8 - 9.0)	0.005
PEEP (cmH_2O)	6 (5 - 8)	8 (5 - 9)	0.251
Peak pressure (cmH_2O)	23 (19 - 29)	23 (20 - 30)	0.631
Plateau pressure (cmH_2O)	21 (18 - 25)	21 (18 - 26)	0.646

Driving pressure (cmH_2O)	14 (10 - 17)	13 (11 - 16)	0.908
Respiratory rate (breaths/min)	18 (15 - 22)	19 (14 - 24)	0.684
**ICU evolution**			
Development of ARDS	20 (8%)	17 (26%)	<0.001
Venoarterial ECMO	11 (2%)	7 (9%)	0.083
Renal replacement therapy	53 (15%)	21 (27%)	0.067
ICU mortality	103 (39%)	26 (39%)	0.671
Causes of death			0.947
Cardiovascular failure	77 (75%)	18 (69%)	
Neurologic failure	14 (13%)	4 (15%)	
Respiratory failure	6 (6%)	2 (8%)	
Other	6 (6%)	2 (8%)	

Table S3. Hazard ratio for mortality of each variable included in the marginal structural model (n=248 unique patients).

	HR	95% confidence interval	p
Driving pressure, 1st week (per cmH_2O)	1.12	1.06 – 1.18	<0.001
Driving pressure, 2+ week (per cmH_2O)	1.06	0.99 – 1.14	0.118
PEEP, 1st week (per cmH_2O)	1.08	0.96 – 1.21	0.204
PEEP, 2+ week (per cmH_2O)	1.15	0.02 – 1.30	0.020
Tidal volume, 1st week (per ml/PBW)	0.84	0.67 – 1.05	0.129
Tidal volume, 2+ week (per ml/PBW)	0.69	0.52 – 0.93	0.015
High-pressure class	0.74	0.40 – 1.38	0.347
PaO_2 / FiO_2 (per unit)	1.00	0.99 – 1	0.079
$PaCO_2$ (per mmHg)	0.99	0.97 – 1.03	0.743
Age (per year)	1.03	1.00 – 1.05	0.060
Male gender	0.74	0.40 – 1.36	0.337
COPD	1.48	0.72 – 3.03	0.284
Chronic kidney failure	0.86	0.39 – 1.87	0.702
Chronic heart failure	1.29	0.70 – 2.39	0.417

Table S4. Hazard ratio for mortality of each variable included in a marginal structural model including only patients under controlled mechanical ventilation on ICU day 1 (N=209).

	HR	95% confidence interval	p
Driving pressure, 1st week (per cmH_2O)	1.11	1.05– 1.18	<0.001
Driving pressure, 2+ week (per cmH_2O)	1.04	0.97 – 1.13	0.264
PEEP, 1st week (per cmH_2O)	1.04	0.91 – 1.19	0.581
PEEP, 2+ week (per cmH_2O)	1.14	0.97 – 1.33	0.103
Tidal volume, 1st week (per ml/PBW)	0.80	0.65 – 0.98	0.032
Tidal volume, 2+ week (per ml/PBW)	0.72	0.54 – 0.96	0.027
Low-pressure class	1.23	0.66 – 2.29	0.522
PaO_2 / FiO_2 (per unit)	1.00	0.99 - 1	0.067
$PaCO_2$ (per mmHg)	1.00	0.97 – 1.03	0.786
Age (per year)	1.02	1.00 – 1.05	0.109
Male gender	0.64	0.32 – 1.29	0.212
COPD	1.37	0.64 – 2.97	0.420
Chronic kidney failure	0.71	0.28 – 1.77	0.459
Chronic heart failure	1.32	0.69 – 2.52	0.406

Table S5. Hazard ratio for mortality of each variable included in a marginal structural model excluding patients in which plateau pressures were imputed (n= 167 patients).

	HR	95% confidence interval	p
Driving pressure, 1st week (per cmH_2O)	1.12	1.04 – 1.20	0.002
Driving pressure, 2+ week (per cmH_2O)	1.01	0.91 – 1.11	0.905
PEEP, 1st week (per cmH_2O)	1.04	0.91 – 1.19	0.588
PEEP, 2+ week (per cmH_2O)	1.49	0.99 – 2.25	0.057
Tidal volume, 1st week (per ml/PBW)	0.81	0.64 – 1.03	0.081
Tidal volume, 2+ week (per ml/PBW)	0.75	0.51 – 1.11	0.156
Low-pressure class	0.72	0.30 – 1.69	0.445
PaO_2 / FiO_2 (per unit)	0.99	0.99 - 1	0.018
$PaCO_2$ (per mmHg)	0.98	0.93 – 1.04	0.540
Age (per year)	1.03	0.99 – 1.07	0.114
Male gender	0.95	0.43 – 2.10	0.896
COPD	1.90	0.74 – 4.91	0.185
Chronic kidney failure	0.73	0.29 – 1.84	0.511
Chronic heart failure	1.03	0.46 – 2.32	0.939

Table S6. Hazard ratio for mortality of each variable included in a marginal structural model excluding patients in which diagnosis of CPE is based only on clinical observations and not supported by any diagnostic technique (n= 229 patients).

	**HR**	**95% confidence interval**	**P**
Driving pressure, 1st week (per cmH_2O)	1.13	1.07 – 1.21	<0.001
Driving pressure, 2+ week (per cmH_2O)	1.17	1.03 – 1.33	0.013
PEEP, 1st week (per cmH_2O)	1.06	0.94 – 1.18	0.331
PEEP, 2+ week (per cmH_2O)	1.12	1.00 – 1.25	0.056
Tidal volume, 1st week (per ml/PBW)	0.87	0.69 – 1.10	0.231
Tidal volume, 2+ week (per ml/PBW)	0.73	0.55 – 0.95	0.022
Low-pressure class	1.47	0.83 – 2.60	0.189
PaO_2 / FiO_2 (per unit)	1.00	0.99 – 1.00	0.176
$PaCO_2$ (per mmHg)	0.99	0.96 – 1.03	0.612
Age (per year)	1.02	0.99 – 1.05	0.091
Male gender	0.87	0.44 – 1.70	0.685
COPD	2.16	1.08 – 4.33	0.030
Chronic kidney failure	1.27	0.58 – 2.80	0.550
Chronic heart failure	1.16	0.63 – 2.12	0.631

Table S7. Hazard ratio for mortality of each variable included in a marginal structural model using dynamic driving pressures (peak inspiratory pressure minus PEEP) (N=287).

	HR	95% confidence interval	p
Driving pressure, 1st week (per cmH_2O)	1.07	1.03 – 1.11	0.001
Driving pressure, 2+ week (per cmH_2O)	1.10	1.02 – 1.19	0.009
PEEP, 1st week (per cmH_2O)	1.09	0.96 – 1.23	0.176
PEEP, 2+ week (per cmH_2O)	1.14	1.02 – 1.28	0.021
Tidal volume, 1st week (per ml/PBW)	0.85	0.68 – 1.05	0.122
Tidal volume, 2+ week (per ml/PBW)	0.68	0.51 – 0.90	0.007
Low-pressure class	0.61	0.35 – 1.06	0.078
PaO_2 / FiO_2 (per unit)	0.99	0.99 - 1	0.014
$PaCO_2$ (per mmHg)	0.99	0.97 – 1.02	0.575
Age (per year)	1.03	1.00 – 1.05	0.019
Male gender	0.69	0.40 – 1.19	0.177
COPD	1.31	0.68 – 2.53	0.423
Chronic kidney failure	0.99	0.49 – 1.99	0.969
Chronic heart failure	1.00	0.58 – 1.73	0.997

Table S8. Hazard ratio for mortality of each variable included in a marginal structural model including patients without invasive mechanical ventilation at ICU admission (n=295).

	**HR**	**95% confidence interval**	**p**
Driving pressure, 1st week (per cmH_2O)	1.10	1.04 – 1.15	<0.001
Driving pressure, 2+ week (per cmH_2O)	0.97	0.89 – 1.05	0.433
PEEP, 1st week (per cmH_2O)	1.07	0.95 – 1.20	0.270
PEEP, 2+ week (per cmH_2O)	1.13	0.99 – 1.30	0.074
Tidal volume, 1st week (per ml/PBW)	0.85	0.69 – 1.05	0.135
Tidal volume, 2+ week (per ml/PBW)	0.75	0.59 – 0.96	0.021
High-pressure class	1.61	0.75 – 3.46	0.224
PaO_2 / FiO_2 (per unit)	0.99	0.99 – 1	0.169
$PaCO_2$ (per mmHg)	0.99	0.96 – 1.02	0.587
Age (per year)	1.01	0.99 – 1.04	0.371
Male gender	0.93	0.52 – 1.69	0.822
COPD	1.86	0.96 – 3.60	0.067
Chronic kidney failure	1.28	0.61 – 2.69	0.513
Chronic heart failure	1.17	0.64 – 2.11	0.611

Figure S1. Hazard ratios (HRs) and 95% confidence intervals (95% CI) of driving pressure obtained in different sensitivity analyses described in supplementary tables 3-8. CPE: Cardiogenic pulmonary edema.

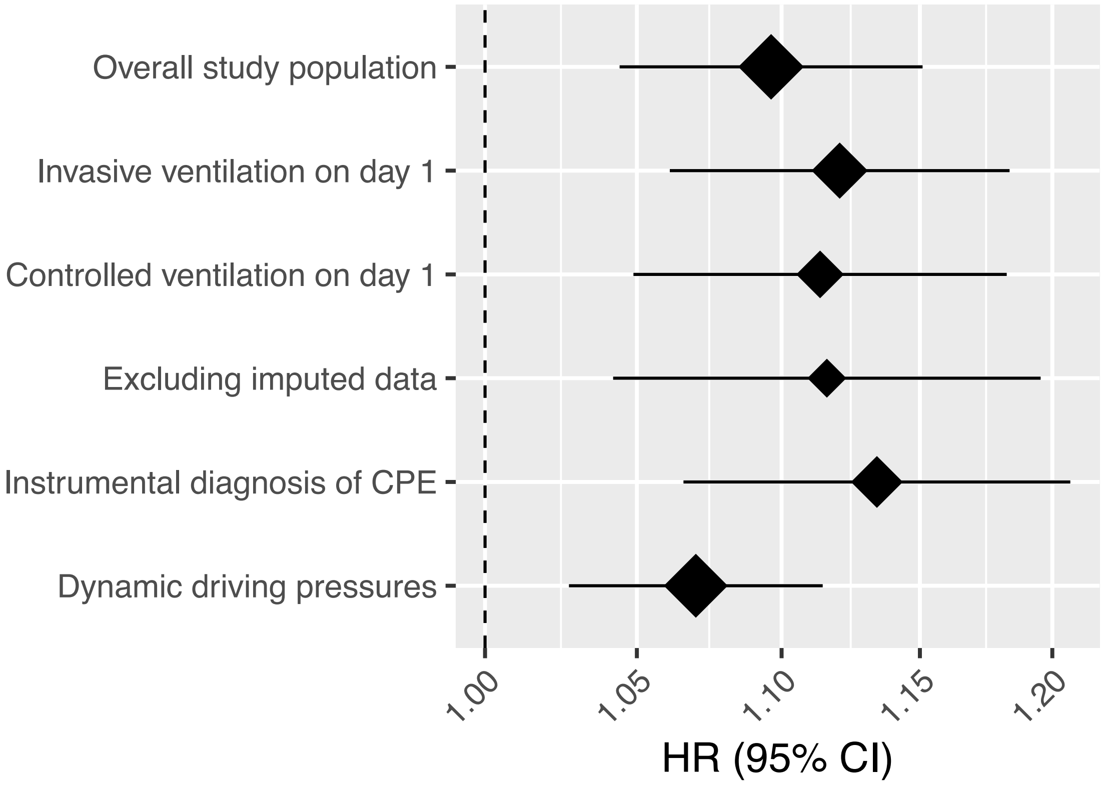

Supplement: Supplementary file 1 — Additional file 1: Table S1. Differences between patients who received non-invasive and invasive ventilation as first-line respiratory support. Table S2. Differences between patients with not prolonged and prolonged invasive mechanical ventilation (more than 10 days). Table S3. Hazard ratio for mortality of each variable included in the marginal structural model (n=248). Table S4. Hazard ratio for mortality of each variable included in a marginal structural model including only patients under controlled mechanical ventilation on ICU day 1 (N=209). Table S5. Hazard ratio for mortality of each variable included in a marginal structural model excluding patients in which plateau pressures were imputed (n= 167 patients). Table S6. Hazard ratio for mortality of each variable included in a marginal structural model excluding patients in which diagnosis of CPE is based only on clinical observations and not supported by any diagnostic technique (n= 229 patients). Table S7. Hazard ratio for mortality of each variable included in a marginal structural model using dynamic driving pressures (peak inspiratory pressure minus PEEP) (n=287). Table S8. Hazard ratio for mortality of each variable included in a marginal structural model including patients without invasive mechanical ventilation at ICU admission (n=295). Figure S1. Hazard ratios (HRs) and 95% confidence intervals (95% CI) of driving pressure obtained in different sensitivity analyses described in Tables S3–S7. CPE: Cardiogenic pulmonary edema. [file 40560_2022_648_MOESM1_ESM.pdf]
